# Germline-targeted baboon apolipoprotein L-1 protects mice against African trypanosomes

**DOI:** 10.1073/pnas.2525773123

**Published:** 2026-03-27

**Authors:** Sara Fresard, Sarah J. Pangburn, Kayla Leiss, Daphne Boodwa-Ko, Daniella Kovacsics, Chris J. Schoenherr, Jeremy S. Rabinowitz, Aris N. Economides, Li Li, Weigang Qiu, Bernardo Gonzalez-Baradat, Alessandro Rosa, Russell Thomson, Jayne Raper, Joseph Verdi

**Affiliations:** ^a^Biology Program, The Graduate Center at the City University of New York, New York City, NY 10016; ^b^Department of Biological Sciences, Hunter College at the City University of New York, New York City, NY 10065; ^c^Regeneron Pharmaceuticals, Tarrytown, NY 10591

**Keywords:** *Trypanosoma brucei*, apolipoprotein l1, trypanosome lytic factor, primate evolution

## Abstract

Some primates are immune to infection by most African trypanosome parasites due to apolipoprotein L-1 (APOL1), a primate-specific ion channel-forming protein. Our long-term objective has been to reduce African trypanosomiasis in livestock by genetic bioengineering of cattle with primate *APOL1*. To select which primate *APOL1*, we analyzed *Papio ssp.* APOL1 proteins and found that *Papio hamadryas APOL1* was a strong candidate for transgenic animal production based on its trypanosome-killing capacity, ion channel properties, and stability. We generated seven transgenic murine lines based on the *P. hamadryas APOL1* sequence and used these mice to investigate the level of *APOL1* expression required for trypanosome immunity in vivo. We challenged the murine lines with three human and four livestock trypanosome isolates. *P. hamadryas APOL1* provided protection against all of the human and three of the livestock trypanosome isolates, though not against *Trypanosoma vivax* despite the logical hypothesis that APOL1 plays a role in primate immunity to that parasite. Occasionally, lower *APOL1* expression in heterozygote mice selected for the emergence of APOL1 resistant parasites in several trypanosome spp. Alarmingly, these resistant parasites were also resistant to high levels of APOL1 in homozygous mice, indicating an increase in virulence. A more-highly expressed chimeric *APOL1* transgene encoding *Homo sapiens APOL1* with the *P. hamadryas APOL1* C-terminus was more effectively protective in heterozygote mice; however, we could not produce homozygous mice, suggesting endogenous toxicity to the mice. Together, these data bear relevance to our long-term objective to generate transgenic APOL1 cattle, the feasibility of which is discussed.

## Significance

Apolipoprotein L-1 (APOL1) provides primates immunity to trypanosome infection. Other animals do not express the protein and are thus susceptible to trypanosomiasis, with cattle in particular routinely succumbing to infection. Fresard et al., generate a panel of APOL1 transgenic mice to assess whether a genetic engineering approach could be deployed as an endemic disease control strategy in livestock, with surprising results.

African trypanosomes proliferate in the bloodstream of mammals after transmission by the tsetse fly. Trypanosome endemicity restricts the agricultural and economic development of the African continent, resulting in three million cattle fatalities per year and subsequent annual losses in the range of 1 to 4 billion United States Dollars (1). There has been a decline in human infection and mortality in recent years (<1,000 reported cases per year) (2), which is attributed to efficient disease control efforts. Notably, there are fundamental genetic elements that underpin human and animal trypanosome resistance and susceptibility; the innate immune protection afforded exclusively to primates by the haptoglobin-related protein (*HPR*) and apolipoprotein L-1 (*APOL1*) genes. HPR and APOL1 are antimicrobial proteins that circulate in plasma on high-density lipoprotein (HDL) complexes called trypanosome lytic factors (TLFs) (3–5). TLFs are endocytosed by the trypanosomes either by fluid-phase or by receptor-mediated uptake. The latter is driven by an interaction between the trypanosome haptoglobin-hemoglobin receptor (HpHbR) and the HPR protein, facilitating APOL1 internalization (6). APOL1 is a channel-forming protein that forms cation-permeable channels in parasite membranes. Channel formation by human APOL1 involves an initial membrane insertion step that requires the acidic pH of the endocytic pathway, followed by pH neutralization-mediated channel opening, which may occur after recycling to the plasma membrane (7–9). This process ultimately leads to osmotic lysis of the trypanosome (10).

HDLs, and therefore TLFs, are synthesized primarily in the liver before being secreted into plasma (11). *HPR* mRNA is produced only in the liver (12). Currently, HPR protein has no hypothesized function outside of TLF and HDL-associated biology. Conversely, *APOL1* mRNA is more ubiquitous and roles of APOL1 have been identified in intracellular viral immunity (13) and kidney function (14). Various kidney cell types express *APOL1* without incident in the majority of the human population. However, a fraction of individuals with recent African ancestry harbor the deleterious *APOL1* variants called G1 or G2 which can lead to a variety of APOL1-associated nephropathies (15). Previous efforts to study these kidney-specific phenotypes in vivo used transgenic (Tg) mice generated by driving the expression of *APOL1* using kidney-specific promoters (16, 17). However, these mice do not produce detectable plasma APOL1 or TLFs. We have previously used a model of hydrodynamics-based gene delivery (HGD) to study HPR and APOL1 function in vivo, in part because the main target of the plasmid injection is the liver, and we could model the function of circulating TLF (18, 19). However, this genetic modification strategy is transient, with expression only lasting about 2 wk. Therefore, there remains a need for a murine model that stably produces primate TLFs.

*Trypanosoma brucei rhodesiense* and *Trypanosoma brucei gambiense* are the only African trypanosomes that regularly infect humans. Human infection by these parasites is mediated by independently evolved APOL1 resistance mechanisms. *T. b. rhodesiense* parasites express serum resistance associated (SRA) protein, which prevents human APOL1 channels from forming by directly binding to the C-terminal domain of APOL1 and inhibiting membrane insertion (5, 19–21). However, SRA does not provide resistance against baboon (*Papio* species) APOL1. Specific amino acids near the baboon APOL1 C-terminus prevent SRA binding in vitro, and transient (HGD) *Papio hamadryas APOL1* expression protects mice from infection by *SRA*-expressing trypanosomes in vivo (21). Transient expression of chimeric *APOL1* proteins that encode the majority of the human *APOL1* sequence fused to the *P. hamadryas* C-terminus also provided protection (21).

The mechanism of human APOL1 resistance by *T. b. gambiense* is a combinatorial mechanism consisting of a mutated HpHb receptor, which reduces APOL1 uptake, and the *Trypanosoma gambiense-*specific glycoprotein (TgsGP) which may block APOL1 membrane insertion (22–24). However, this resistance mechanism is not sufficient to protect against lysis by *Papio papio* APOL1 (25). The molecular mechanism by which some *Papio spp.* APOL1 channels bypass the function of TgsGP and lyse *T. b. gambiense* has not yet been characterized (26).

Published literature suggests differential susceptibility of various *Papio* spp. to *T. b. gambiense*. *P. papio* serum lyses *T. b. gambiense* in vitro(25) and *P. papio* and *P. hamadryas* are reported to be resistant to *T. b. gambiense* infection in vivo (27). However, *Papio cynocephalus* serum did not lyse *T. b. gambiense*in vitro (28) and *P. cynocephalus* are susceptible to *T. b. gambiense* in vivo (29). Whether this differential resistance to *T. b. gambiense* infection can be attributed to the APOL1 protein remains unclear as many of these experiments were performed prior to the discovery of APOL1, and to date, only three of six *Papio APOL1* gene sequences are available (one each from *P. papio*, *P. hamadryas*, and *Papio anubis*).

Here, we used whole genome sequencing data generated by the Baboon Genome Consortium (30) to expand the number of available *Papio APOL1* sequences to 33, representing all six species. We then characterized the trypanolytic potential of the most common APOL1 variants specific to *P. papio*, *P. hamadryas*, and *P. cynocephalus,* revealing significant changes in protein function dependent on species-specific polymorphisms. We show that *P. hamadryas* APOL1, similar to *P. papio* APOL1, can kill *T. b. gambiense* parasites and subsequently followed up on the existing characterization of *P. hamadryas* channel biology (7) with additional electrophysiological studies. A panel of genetically modified mouse lines were engineered to produce optimally trypanolytic *APOL1* variants with or without *P. hamadryas HPR* coexpression, providing useful resources to study the interactions between HDL-associated APOL1 and a broad range of pathogens. These experiments reveal notable differences in the dynamics of infection by a variety of trypanosome species, including *T. b. gambiense, T. b. rhodesiense, Trypanosoma evansi, Trypanosoma congolense, and Trypanosoma vivax*. In particular, *T. vivax*, which is responsible for cattle infection along with *Trypanosoma* *brucei* and *T. congolense*, is not recognized as being human infective. Despite the logical hypothesis that APOL1 plays a role, it has not been confirmed as the mechanism of human immunity (31). We provide data that do not support this hypothesis and instead reveals that mice that express *APOL1* and are protected against *T. brucei* remain susceptible to *T. vivax*. This bears particular relevance to our long-term goal of assessing whether genetically engineered *APOL1*-expressing livestock could provide an additional layer of disease control in Africa, the feasibility of which is discussed.

## Results

### Baboon *APOL1* Is Polymorphic within and across *Papio* Species.

**Fig. 1.**
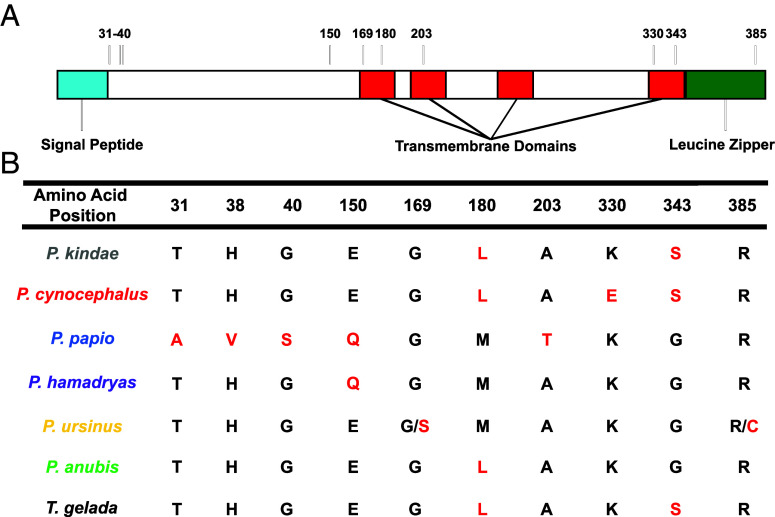
The consensus sequences of each *Papio* species APOL1 protein. (*A*) Illustration of the putative domain architecture of APOL1 (determined using JPred4), highlighting the location of polymorphic sites that differentiate the consensus sequences of the species. APOL1 has an N-terminal signal peptide (aqua), four predicted membrane spanning helices (JPred4, red), and a C-terminal leucine zipper motif (green). (*B*) Only the polymorphic amino acids that define the consensus sequences are listed, meaning that every nonlisted amino acid position is identical between all species consensus sequences. Polymorphic sites specific to certain subgroups are listed in *SI Appendix*, Fig. S1. Sites that differ from the consensus among the different species are highlighted in red. Positions 169 and 385 in *Papio ursinus* APOL1 cannot be assigned given the low number of individuals (n = 2), so both possible variants are listed.

The *APOL1* gene displays evidence of positive selection in primates (32, 33). We investigated whether the *Papio spp. APOL1* gene displayed similar patterns of selection. To determine whether *APOL1* polymorphisms exist within the lineage, we analyzed data collected by the Baboon Genome Consortium. By analyzing data from 15 individual baboons (samples listed in *SI Appendix*, Table S1) representing all six *Papio* species, we identified 39 polymorphic sites within the five exons of *APOL1*. These sites were used to construct haplotypes and a phylogenetic tree rooted by a closely related outgroup, the gelada baboon (*Theropithecus gelada*) (*SI Appendix*, Fig. S1). Many individuals had insertions and deletions (indels) within their *APOL1* sequences, which we have separately summarized for clarity at the protein level (*SI Appendix*, Table S2). The species-specific consensus sequences of the proteins are listed in Fig. 1. To query whether *Papio APOL1* is under positive selection, as has been determined across primate genera (33), we quantitated the ratio of nonsynonymous polymorphisms (dN) to synonymous polymorphisms (dS) within the gene (*SI Appendix*, Table S3). The dN/dS ratio was estimated to be 0.5773 under a neutral model using maximum likelihood. When allowing dN/dS to vary along the sequence, 74.9% of the sites showed dN/dS = 0, 25.1% of the sites showed dN/dS = 1, and no sites showed dN/dS > 1. While the likelihood ratio test did not statistically support the positive selection model, the single-ratio estimate of dN/dS = 0.5773 is on par with the dN/dS ratio of immune-related proteins in humans (34).

### *P. cynocephalus* APOL1 Is Less Trypanolytic Than Other *Papio* APOL1 Proteins.

**Fig. 2.**
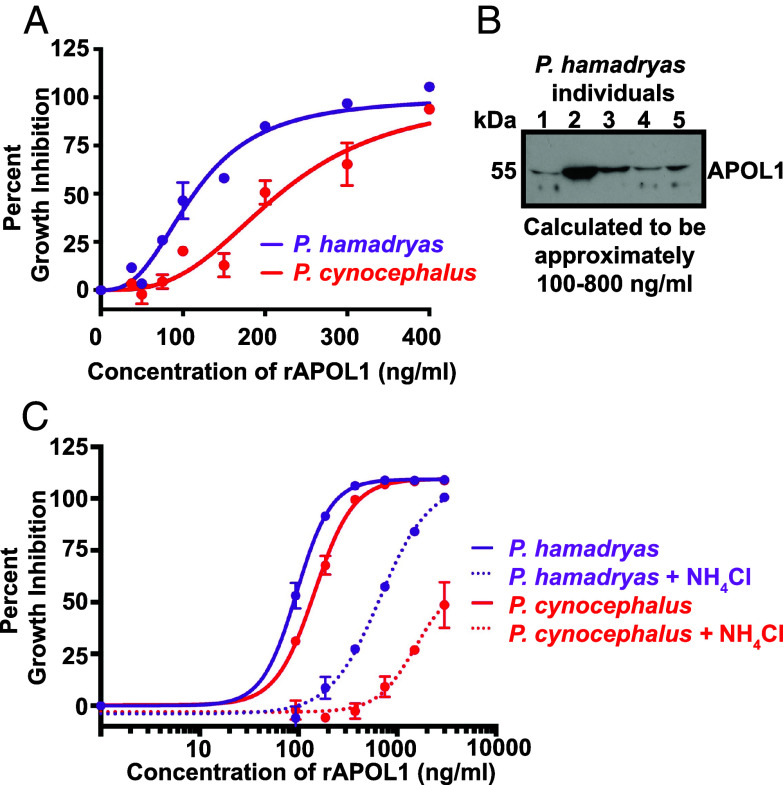
Baboon *APOL1* polymorphisms modulate the trypanolytic capacity of the protein (*A*) 24-h trypanolysis assay showing the lytic capacity of serially diluted *P. hamadryas* and *P. cynocephalus* rAPOL1 proteins against *T. b. brucei* parasites. (*B*) Individual *P. hamadryas* plasma samples reveal the range of concentrations of APOL1. The band intensities compared to a serially diluted sample of rAPOL1 via pixel counts using Fiji/ImageJ. (*C*) 24-h trypanolysis assay showing lytic capacity of serially diluted *P. hamadryas* and *P. cynocephalus* rAPOL1 under control conditions (solid line) and when the endosomal system has been neutralized (dotted lines) using 20 mM NH_4_Cl treatment of the parasites 30 min prior to APOL1 treatment.

*P. papio* APOL1 synthesized recombinantly in bacteria (rAPOL1) kills *T. b. gambiense* parasites in vitro (25), and *P. hamadryas* are refractory to *T. b. gambiense* infection in vivo (27). *P. cynocephalus*, however, are susceptible to *T. b. gambiense* infection (29). *P. cynocephalus* serum could not kill *T. b. gambiense* parasites in vitro (28), and the *P. cynocephalus APOL1* variants were divergent from the *P. hamadryas* and *P. papio APOL1* sequences in our phylogenetic analysis (Fig. 1). To determine if the ability to kill *T. b. gambiense* was restricted to *P. papio* APOL1, we synthesized recombinant *P. papio*, *P. hamadryas, and P. cynocephalus* APOL1 variants using *Escherichia* *coli* (*SI Appendix*, Fig. S2 *A* and *B*) and performed in vitro trypanosome lytic assays. Like human APOL1, all three *Papio* proteins killed *T. b. brucei*, however, unlike human APOL1, only the *Papio* proteins killed *T. b. gambiense* (*SI Appendix*, Fig. S2*C*), suggesting that the ability to lyse *T. b. gambiense* may be afforded to all *Papio* APOL1s. In our studies, we found that *P. cynocephalus* APOL1 was less lytic to trypanosomes than *P. hamadryas* (Fig. 2*A*), but still able to kill *T. b. gambiense* at higher concentrations (*SI Appendix*, Fig. S2*C*). To determine if the concentrations required to kill trypanosomes were physiologically relevant, we quantified the circulating plasma-APOL1 concentrations in a panel of *P. hamadryas* samples (Fig. 2*B*). *P. hamadryas* plasma-APOL1 levels range from 100 to 800 ng of protein per mL of plasma (Fig. 2*B*). The concentrations of rAPOL1 needed to kill trypanosomes fell within this range of protein concentration (Fig. 2*A*). Weak bases, such as ammonium chloride, inhibit trypanolysis by human APOL1. In contrast, we observed that while ammonium chloride treatment increased the amount of *P. hamadryas* and *P. cynocephalus* APOL1 needed to kill the trypanosomes, it was not sufficient to completely block activity (Fig. 2*C*). This suggests that the balance between *T. b. gambiense* infection and immunity in *Papio spp.* is likely multifactorial, involving species-specific amino acid residues (Fig. 1), differences in plasma-APOL1 levels (Fig. 2*B*), and differences in pH regulation (Fig. 2*C*) that are required for optimal APOL1 function.

### *Papio* APOL1 Proteins Form Ion Channels in Planar Lipid Bilayers.

**Fig. 3.**
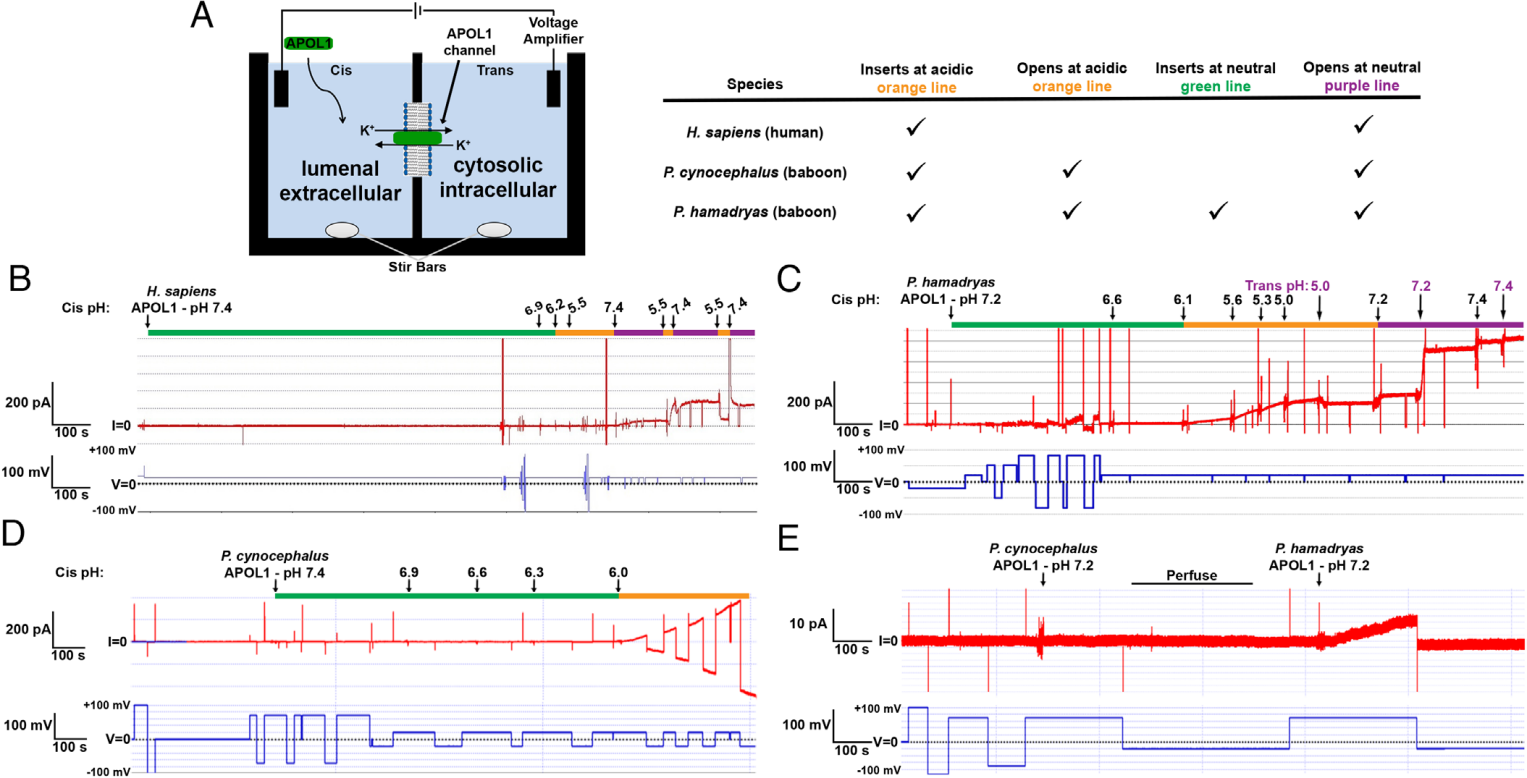
Differential channel formation by *Papio* APOL1 proteins. (*A*) Schematic illustrating the planar lipid bilayer. Two compartments containing 1 mL buffered potassium chloride (bilayer buffer; see *Methods*) are separated by a phospholipid and cholesterol bilayer. APOL1 is added to the “Cis” side, while the opposite side is the “Trans” side. Voltage is set to mimic a membrane potential (blue line) The pH is set by adding precalibrated volumes of potassium hydroxide or hydrochloric acid to the continuously stirred solutions. Formation of ion-permeant channels in the bilayer is indicated by electrical current (I, red line). A table summarizing the pHs at which each protein can insert and open is on the right. (*B–E*) The voltage (in millivolts) is shown in blue in the lower traces, and the current output (in picoamperes, pA) is shown in red in the upper traces. The green lines indicate neutral pH, prior to acidification. The orange lines show acidification. The purple lines indicate neutralization after acidification. (*B*) The channel forming capacity of *Homo sapiens*. (*C*) The channel forming capacity of *P. hamadryas* APOL1 at Cis (black) and Trans (purple) pH values. (*D*) The channel forming capacity of *P. cynocephalus* APOL1 at different Cis pH values. (*E*) The channel forming capacities of *P. cynocephalus* APOL1 and *P. hamadryas* APOL1 at neutral pH on the same bilayer. During the period marked with the black bar (labeled as “Perfuse”) the cis compartment was perfused with chamber buffer (pH 7.2) to remove non-membrane-associated protein.

We hypothesized that the difference in lytic capacity between baboon APOL1 variants involved differences in the channel-forming mechanisms of human and baboon APOL1 proteins. We proceeded to characterize *P. hamadryas* and *P. cynocephalus* APOL1 in planar lipid bilayers (Fig. 3*A*, data summarized in the paired table). This artificial bilayer is given a membrane potential (lower trace in Fig. 3 *B–E*, in blue, measured in millivolts), which can be switched from positive to negative to ensure membrane stability and affects the direction of ion movement. When a channel is inserted and open, there is a passage of ions measured as current (top trace in red, measured in picoamps). In each experiment, most pH modifications are made on the Cis side (Fig. 3*A*); APOL1 is added to the cis side at a neutral pH (green line on top of the trace). The pH is then acidified (orange line) to mimic the acidic endosome, and then neutralized (purple line) to mimic the recycling of endosomal membrane to the plasma membrane. The trans side mimics the cytoplasm in all instances, and is maintained at pH 7.4 unless otherwise stated.

Human APOL1 associates with planar lipid bilayers at an acidic pH (Fig. 3*B*, orange line), inserting but remaining as a closed channel, and proceeds to form open cation-selective channels only when the pH is neutralized (9) (Fig. 3*B*, purple line). In contrast, *P. hamadryas* APOL1 begins to associate with planar membranes at neutral pH (7) (Fig. 3*C*, green line), allowing ion flow. Additional membrane insertion and/or opening is stimulated by Cis acidification (Fig. 3*C*, orange line), and the current then increases approximately threefold upon subsequent Cis neutralization (Fig. 3*C*, purple line). Similar to human APOL1 (9), *P. hamadryas* APOL1 channel conductance is maximal when the environments on both sides of the channel are neutral (Fig. 3*C*, purple line). These data indicate that *P. hamadryas* APOL1 forms channels through a less pH-restricted process than human APOL1 and agrees with previously published results (7). We then characterized the pH dependence of the *P. cynocephalus* APOL1 variant and observed that, similarly to *P. hamadryas* APOL1, the protein can form conductive channels after encountering an acidic pH (Fig. 3*D*, orange line), although the protein did not permit detectable ion flux at neutral pH prior to acidification (Fig. 3*D*, green line). To confirm these results, we analyzed the channel forming capacity of both variants on the same membrane at neutral pH and observed that only *P. hamadryas* APOL1 produced a detectable ion flux (Fig. 3*E*). Together, this shows that these two *Papio* APOL1s are less restricted by pH than human APOL1, as they are both able to open channels and permit cation flux at an acidic pH, unlike human APOL1. Only *P. hamadryas* APOL1, however, can insert at a neutral pH. This suggests that the increased lytic capacity of *P. hamadryas* APOL1 (Fig. 2 *A* and *C*) could be a function of less restricted channel formation relative to *P. cynocephalus* APOL1. This likely contributes to trypanosome immunity in vivo.

### Integration of the Genomic *P. Hamadryas APOL1* Locus into the Mouse Genome Does Not Protect from Trypanosome Infection.

Baboon and human *APOL1* are predicted to encode a 42 kDa protein. However, *P. hamadryas* APOL1 migrates at 55 kDa on SDS-PAGE gels, suggesting that it undergoes posttranslational modification. Peptide *N*-glycosidase F (PNGase F) treatment of *P. hamadryas* HDL resulted in a gel mobility shift in APOL1, revealing that the protein is *N*-glycosylated (*SI Appendix*, Fig. S3). However, deglycosylation does not produce the expected 42 kDa human APOL1-sized protein, suggesting that other posttranslational modifications (PTMs) are present. Importantly, all previous reports directly showing that *Papio* APOL1 proteins are capable of lysing trypanosomes have used subphysiological levels of *Papio* serum or recombinant APOL1 isolated from *E. coli*, which lack the enzymes required for the addition of N-linked glycans (35).

In order to investigate the function of baboon APOL1 in a physiologically relevant manner, we generated a panel of genetically modified mice expressing baboon *APOL1* utilizing different combinations of promoters and constructs. To build our first construct, we obtained a 20 kilobase segment of the *P. anubis* genome in a bacterial artificial chromosome (BAC) from the Children’s Hospital Oakland Research Institute. The BAC contained all five *APOL1* exons, the putative endogenous promoter, and the upstream and downstream untranslated regions of the gene which were predicted to contain the endogenous regulatory regions required for expression. This *P. anubis* DNA was isolated from the individual animal used for the baboon reference genome Panu2.0, which differs from the *P. anubis APOL1* consensus sequence at two positions. We inserted this *P. anubis APOL1* construct into the *ROSA26* locus (*SI Appendix*, Fig. S4*A*), a ubiquitously euchromatic region of the genome commonly used for transgene placement in mice. In another construct, we generated a version of the BAC that encoded a *P. hamadryas APOL1* gene by inserting two separate base pair changes that encoded the E150Q and L180M substitutions (Fig. 1). The *P. hamadryas APOL1* construct was inserted into the mouse genome immediately upstream of the *myosin heavy chain 9* gene (*SI Appendix*, Fig. S4*B*), which is next to the endogenous location of *APOL1* in primates. We tested whether the germline Tg mice could express and secrete APOL1, and could not detect plasma or HDL-associated APOL1. We detected *APOL1* RNA production in liver lysates (*SI Appendix*, Fig. S4*C*), suggesting that the loci were transcriptionally active. The mice were not protected from infection by *T. b. brucei* parasites (*SI Appendix*, Fig. S4*D*). This was consistent with previously published results using transiently Tg animals in which *P. hamadryas* APOL1 expression could only clear a trypanosome infection in mice that also expressed human APOA-I (19, 36). Therefore, we crossed our Tg *APOL1*-expressing mice with human *APOA-I*-expressing mice, to potentially generate HDLs carrying both human APOA-I and *P. anubis* APOL1. However, these double Tg mice were also susceptible to infection by *T. b. brucei* (*SI Appendix*, Fig. S4*E*). Coexpressing *P. hamadryas HPR* by HGD in the *P. anubis APOL1*-expressing mice (*SI Appendix*, Fig. S8*A*) was able to provide a slight but not statistically significant level of protection from *T. b. brucei* infection (*SI Appendix*, Fig. S4*F*). This suggests that although we were unable to detect any APOL1 protein in plasma or HDL, there may be a small amount of circulating and functional APOL1 protein in the sera of these mice stabilized by HPR. Nevertheless, we concluded that this genetic modification strategy did not produce a suitable model system.

### Stable Expression of the *P. Hamadryas APOL1* CDNA Protects Mice from Trypanosome Infection.

**Fig. 4.**
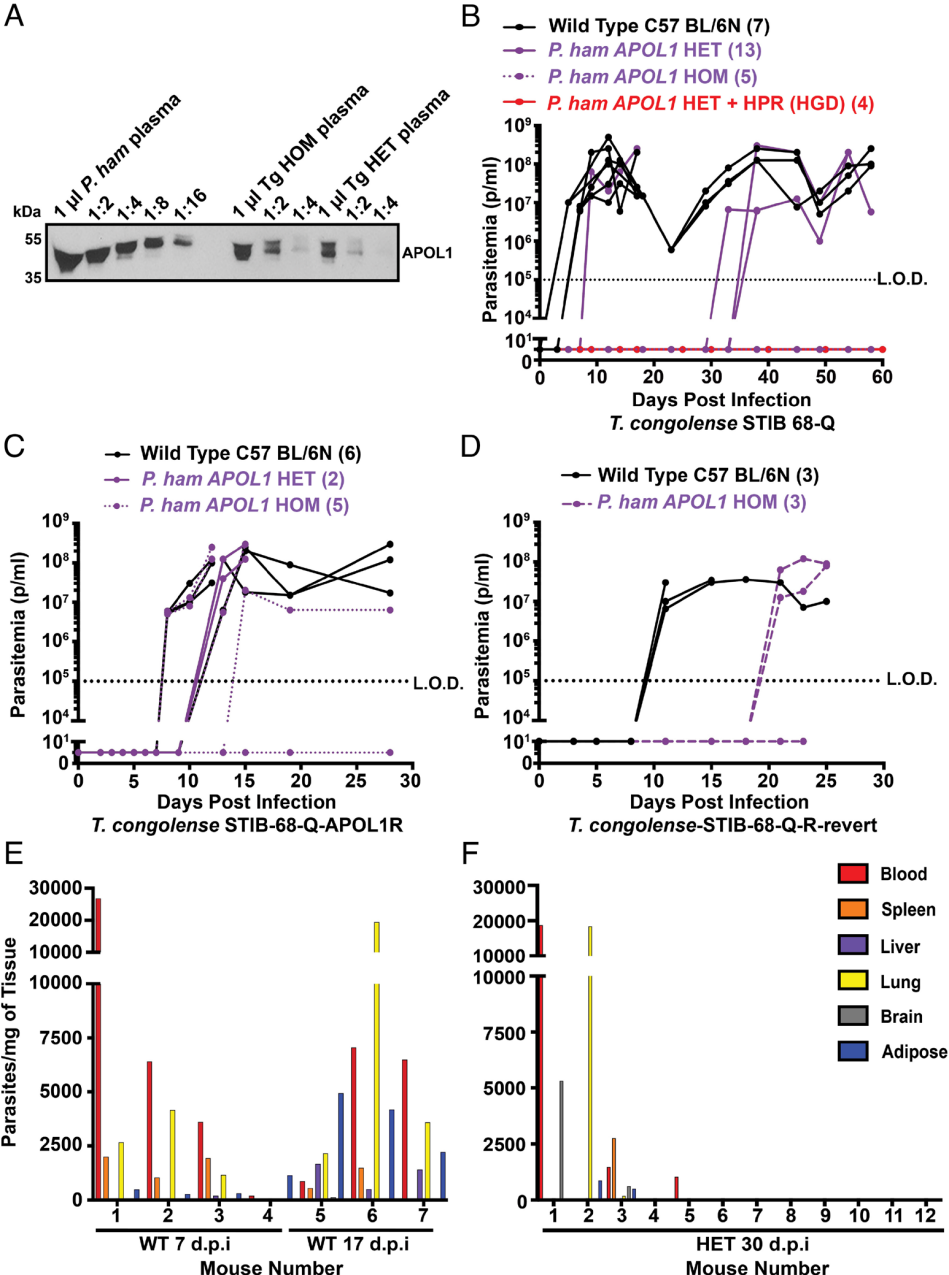
Low plasma concentration of APOL1 selects for APOL1-resistant *T. congolense*. (*A*) Anti-baboon APOL1 western blot of serially diluted *P. hamadryas* (*P. ham*) plasma and Tg HOM and HET mouse plasma samples, starting with 1 uL as the maximal plasma load per sample followed by twofold serial dilution. (*B*) Mice were inoculated with 5,000 parasites of *T. congolense* and parasitemia was monitored by light microscopy at least every 3 d. Mice were infected 2 d after plasmid injection by HGD. Parasitemia over time showing the number of parasites per ml of blood in Tg mice inoculated with 5,000 pleomorphic *T. congolense* (STIB68-Q) parasites by i.p. on Day 0. The graph shows parasitemia in each individual mouse of each genotype (number of mice per genotype in parentheses). The data are presented this way because these are pleomorphic parasites, which do not produce Kaplan Meyer curves that are reliably interpretable. Each line is one mouse. The limit of detection is approximately 1 × 10^5^ parasites per mL (p/mL) (denoted with black dotted line, L.O.D.). Mice were removed from the experiment when parasitemia reached 1 × 10^8^ p/mL indicated by the line ending at that time point. (*C*) Challenge with STIB 68-Q-APOL1R was derived from a heterozygote mouse infected with *T. congolense* STIB 68-Q that developed a sustained parasitemia. (*D*) Challenge with the STIB 68-Q-APOL1R-revert strain, which was derived from a STIB 68-Q-APOL1R parasite population that was passed through a wild type (WT) mouse for 1 mo. (*E* and *F*) The calculated number of parasites per milligram of perfused (all blood removed) tissue in WT C57 BL/6N-J mice (*E*) or HET *P. hamadryas APOL1*-Tg mice (*F*) after DNA isolation and qPCR using primers specific to *T. congolense Cathepsin L* (*CATL*) (housekeeping gene) compared to a standard curve of a known number of parasites. Each number denotes one individual mouse. D.P.I.; Days Postinfection.

We next proceeded to target the *ROSA26* locus with a more controlled *APOL1* expression system by integrating the cDNA of *P. hamadryas APOL1* under the control of a mouse-compatible ubiquitin promoter (*SI Appendix*, Fig. S5*A*) to attempt to generate transgene expression in a physiological manner, given that *APOL1* is expressed in a wide variety of cells, similar to ubiquitin (12). We chose *P. hamadryas* because this protein’s function is the most extensively characterized in the literature from the channel biology (7) and in vivo expression perspective (21, 36), and because we had observed that it was capable of lysing *T. b. gambiense* parasites similar to *P. papio* APOL1 (*SI Appendix*, Fig. S2*C*). We used cDNA to reduce the size and complexity of the construct for improved cloning efficiency. These mice produced plasma circulating APOL1 protein, albeit approximately 8- to 16-fold less than an average *P. hamadryas baboon* in terms of protein per mL of plasma, with the homozygous (HOM) mice producing approximately twice as much protein as heterozygous (HET) mice (Fig. 4*A*). Importantly, the APOL1 in the mouse plasma and *P. hamadryas* plasma are the same molecular weight, suggesting that the mice posttranslationally modify the protein physiologically (Fig. 4*A*). The APOL1 was present on HDL isolated from mice, thus forming a TLF-like complex (*SI Appendix*, Fig. S5*B*). To test whether the mice were protected from *T. b. brucei* infection, we infected the mice with 5,000 *T. b. brucei* parasites. An inoculum of 5,000 parasites is in the range of parasites transmitted by an individual tsetse bite, although the range is very large and not well defined (0 to 40,000 cells per bite; mean of 3,200) (37). With this inoculum of parasites, the *APOL1*-expressing mice were fully protected from infection (Table 1 and *SI Appendix*, Fig. S5*C*). Notably, this protection was achieved in mice expressing murine APOA-I, contrary to the previously published data using transiently Tg mice that required human APOA-I (19, 36).

**Table 1.** Summary of Tg mouse constructs and protection against Trypanosome species


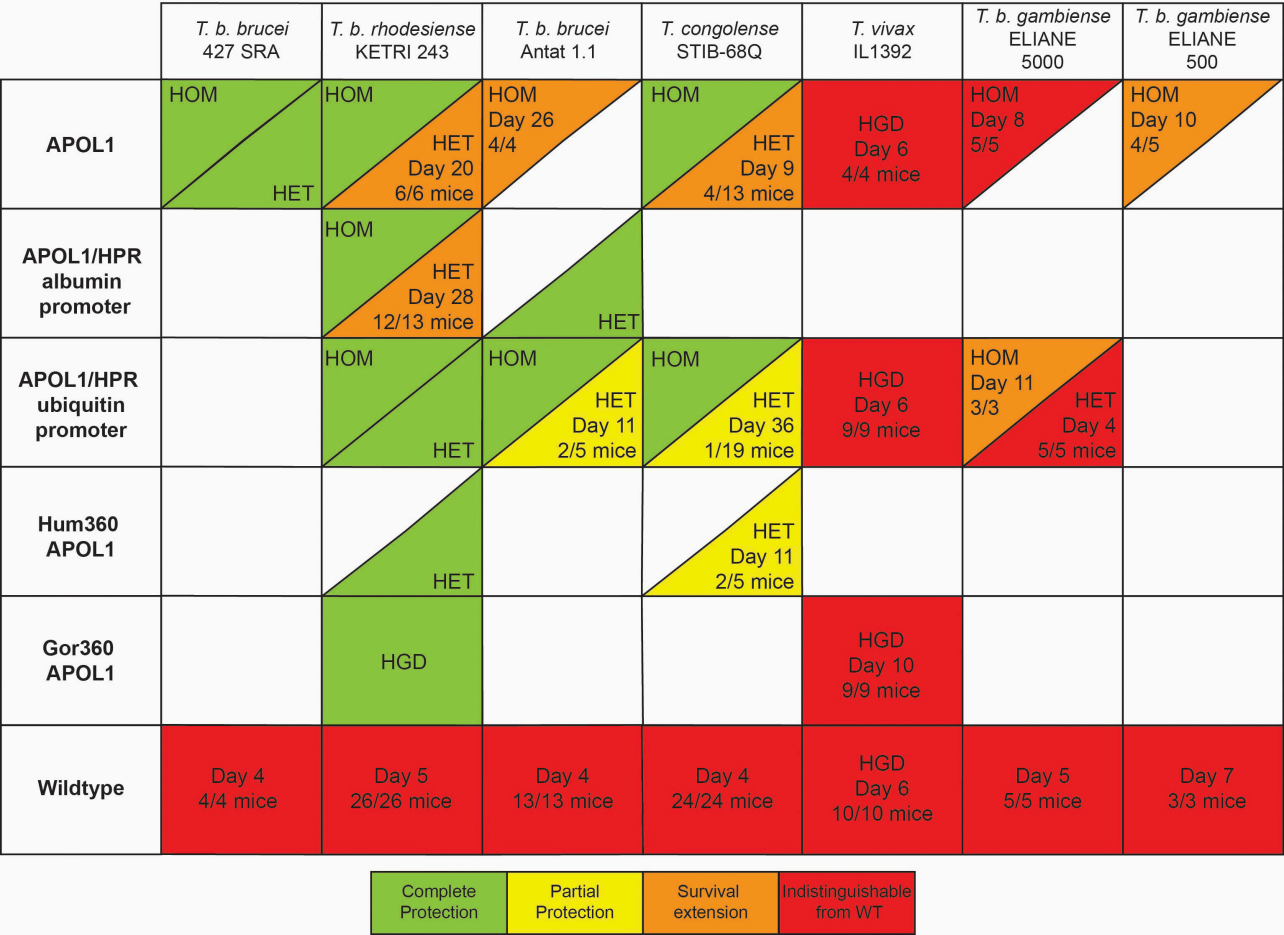

Mouse genotypes are indicated on the left, with each parasite tested on top. Where germline Tg animals were tested, a line across the box is used to distinguish between HOM and HET mice. Transient Tg animals had the gene introduced by hydrodynamic gene delivery (HGD), and mice were infected 2 d later. If mice developed parasitemia, the first day a mouse had detectable parasites is denoted, followed by the total number of mice that developed parasites (“HET, Day 15, 8/8 mice” means of the eight HET mice infected, all eight developed parasitemia, with the first mouse having detectable parasites on day 15 postinfection). Green is complete protection, yellow is partial protection (most mice did not have parasites, and development of parasitemia was delayed), orange is survival extension (all/most mice develop parasites, but mice survived longer than WT counterparts), red is indistinguishable from WT (addition of APOL1 provided no protection at all).

We next assessed general animal welfare of the Tg lines. We used multiple litters of mice to test whether the *APOL1* expression had any apparent negative impact on early postbirth development. We observed that the mice gained weight comparable to WT counterparts (*SI Appendix*, Fig. S5*D*) and exhibited normal behavior. In humans, the G1 and G2 APOL1 variants can cause chronic kidney diseases in adults where the source of damage is locally produced APOL1 lysing the cells that produce it (38), A common manifestation of which is focal segmental glomerulosclerosis (FSGS) (14). As FSGS progresses, the glomeruli of the kidney begin to decay over time as progressive glomerular cell death leads to the formation of scar tissue. Histologically, the glomeruli resect from the walls of the Bowman’s capsule, losing their approximately spherical shape. Importantly, glomerular damage has also been observed in mice expressing the nephrotoxic human *APOL1* variants, using either kidney-specific promoters (16) or the entire genomic region of *APOL1* (39). We therefore prepared kidney tissue sections from adult (greater than 1 y old) Tg *P. hamadryas APOL1* and WT mice for basic histological analyses and observed that the glomeruli in the Tg mice showed no obvious signs of resection and instead resembled WT glomeruli (*SI Appendix*, Fig. S5*E*). We concluded that in this mouse line, *P. hamadryas* APOL1 did not induce any detectable negative outcomes in the tested phenotypes.

Once we determined that *P. hamadryas APOL1* expression caused no obvious deleterious effects, and provided protection from infection by *T. b. brucei*, we proceeded to characterize the protective potential of *P. hamadryas APOL1* expression in the context of a wide range of human-infective and livestock-infective African trypanosomes. Similar to *T. b. brucei* (Table 1 and *SI Appendix*, Fig. S5*C*), the *APOL1*-expressing homozygote mice were fully protected from infection by the globally distributed livestock pathogen *T. b. evansi* (*SI Appendix*, Table S4). After infecting mice with 5,000 *T. b. gambiense* parasites, we found that HOM mice were partially protected from infection (Table 1 and *SI Appendix*, Table S4). Inoculating HOM mice with only 500 parasites extended the survival of the mice significantly, although they still were not fully protected (Table 1 and *SI Appendix*, Table S4). We then investigated whether the mice were protected from infection by *T. b. rhodesiense*, a zoonotic pathogen that infects both humans and cattle. HET mice infected with *T. b. rhodesiense* were partially protected, with a survival extension of approximately 2 to 3 wk relative to WT controls (Table 1 and *SI Appendix*, Table S4). HOM *APOL1*-expressing mice were fully protected from infection (Table 1 and *SI Appendix*, Table S4), suggesting that APOL1 levels are an important determining factor that dictates whether an animal will be fully resistant to infection. We were also able to achieve nearly complete protection by coexpression of *HPR* via HGD in the HET mice (Table 1 and *SI Appendix*, Fig. S8*A* and  Table S4), affirming that TLFs (containing APOL1, HPR, APOA-I) that can be endocytosed by the HpHbR are more effective than TLFs (containing APOL1, APOA-l; lacking HPR) that must be taken up by fluid-phase endocytosis at the same concentration.

We then investigated whether the mice were protected from infection by the livestock infective *T. congolense*. This strain has a variable effect on WT mice, causing chronic infections rather than acute. Therefore, instead of monitoring survival, we periodically measured parasitemia in the mice (Fig. 4*B*). One individual heterozygote *APOL1*-expressing mouse developed a high parasitemia and subsequently died within the first 2 wk of infection, although this was an isolated result that was never replicated. More reproducibly, we observed that approximately 25% of the HET *APOL1*-expressing mice developed a high and sustained parasitemia approximately 5 to 6 wk after infection (Fig. 4*B*). This was never observed in HOM *APOL1*-expressing mice or *APOL1* and *HPR*-expressing mice (Fig. 4*B*), consistent with previous data using *T. b. rhodesiense* (*SI Appendix*, Table S4).

### Previously APOL1-Susceptible Trypanosomes Can Develop APOL1 Resistance in Animals with Sublethal Levels of APOL1.

The *T. b. gambiense*, *T. b. rhodesiense*, and *T. congolense* parasites that grow and survive in the Tg mice are under continuous pressure from the *P. hamadryas* TLF and therefore may represent parasites that have acquired APOL1 resistance. We investigated this phenotype using *T. congolense* by harvesting parasites from a heterozygote mouse that developed a sustained detectable level of parasitemia after an initial infection, and reinfecting those parasites (*T. congolense* STIB 68-Q-APOL1R) into naïve *APOL1*-expressing mice. We observed that the HET and the majority (80%) of the HOM *APOL1*-expressing mice infected with this derived strain of *T. congolense* developed a high parasitemia within the first 2 wk of infection (Fig. 4*C*). We therefore concluded that the parasites had indeed developed APOL1 resistance, either through selection of preexisting minor populations of resistant parasites or de novo mutation(s).

APOL1-resistant *T. congolense* displayed a slowed growth phenotype (reaching peak parasitemia in 8 to 12 d) compared to WT parasites (5 d). We hypothesized that the APOL1 resistance was associated with a fitness cost, and parasites would therefore lose resistance in the absence of the selection pressure mediated by APOL1. To investigate this, we infected WT mice with the resistant parasites (*T. congolense* STIB 68-Q-APOL1R). We harvested the parasites 1-mo postinfection, hypothetically long enough for the resistant population to be outgrown by APOL1-susceptible revertants. These parasites (*T. congolense* STIB 68-Q-APOL1R-revert) were then reinfected into WT mice and HOM *APOL1* mice, revealing that HOM mice were once again resistant to the infection, albeit only partially, with approximately a 2-wk delay in the onset of detectable parasitemia relative to WT mice (Fig. 4*D*). We have not determined the genetic source of the resistance, although this revertible phenotype is reminiscent of the SRA-mediated APOL1 resistance mechanism acquired by *T. b. rhodesiense* which is also readily “turned off” in the absence of APOL1 selective pressure (40).

We then investigated where resistant parasites may have emerged in the *APOL1* HET mice. HDLs circulate in plasma and permeate extravascular tissue spaces. The concentration of HDL in those tissue spaces is approximately 10-fold lower than the concentration that circulates in plasma (41). Since *T. b. brucei* invades and adapts to extravascular tissue spaces (42), we hypothesized that APOL1-resistant *T. congolense* clones could emerge from tissue-resident trypanosomes exposed to this sublethal level of TLF. We observed that during the early stage of infection WT mice, there were a relatively small but detectable number of parasites in the tissues, and that the proportion of tissue-resident parasites increased as the infection progressed (Fig. 4*E*). Of the analyzed tissues, we observed that the adipose tissue and the lung were the most frequently enriched tissues (Fig. 4*E*), suggesting that the parasites may have particular tropism for different tissues. Enrichment in both tissues was more evident at the later stage of the infection (17 dpi).

We then investigated whether there were tissue-resident parasites in *APOL1*-expressing mice. We infected 12 HET mice and harvested tissues at 30 d postinfection, hypothetically immediately preceding any visually detectable parasitemia (based on the data from Fig. 4*B*). We detected parasites in four of the mice, with one mouse (Mouse 2) displaying parasites exclusively in the lung and adipose tissue, with no detectable blood-resident parasites (Fig. 4*F*). Notably, no parasites were ever detected in HOM *APOL1*-expressing mice. These data suggest that tissues could provide a niche with sublethal levels of APOL1 that selects for the emergence of resistance.

### Coexpression of *HPR* and *APOL1* Augments Protection against Trypanosome Infection.

**Fig. 5.**
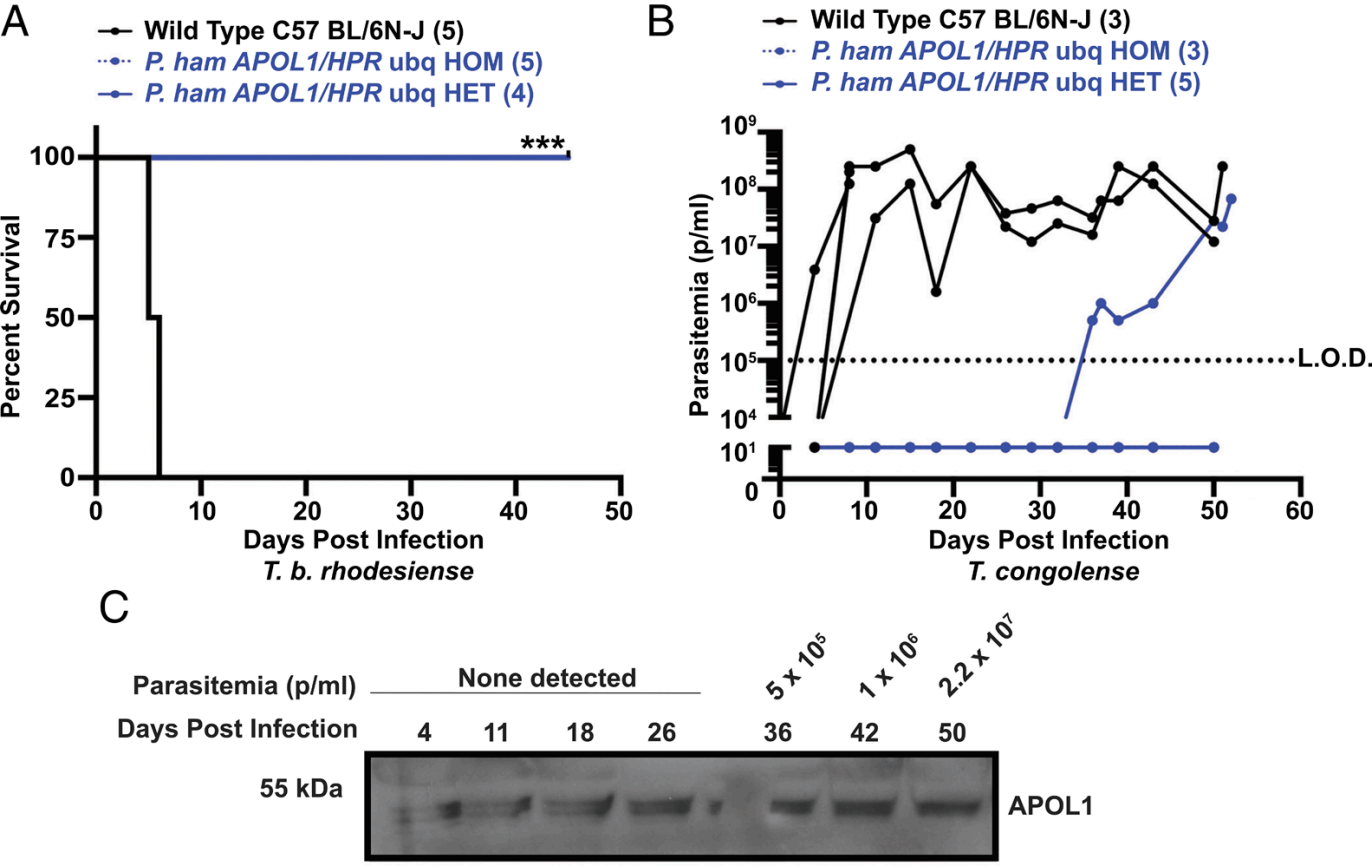
Targeted integration of *P. hamadryas APOL1* and *HPR* is not sufficient to mediate sterile protection against trypanosome infections (*A*) Kaplan–Meier survival curve of WT, HET, and HOM infected with *T. b. rhodesiense* (KETRI243) (***P = 0.0010; Log-rank test). (*B*) *T. congolense (STIB-68Q)* parasite challenge in mice WT, HET, and HOM for *APOL1* and *HPR*, both on ubiquitin promoters. Parasitemia over time showing the number of parasites per mL of blood in each individual Tg mice inoculated with 5,000 pleomorphic *T. congolense* (STIB68-Q) parasites by i.p. on day 0. The data are presented this way because these are pleomorphic parasites, the observations from which are best observed by following parasitemia onset and duration. Each line is one mouse. Parasitemia was counted by light microscopy; the limit of detection is approximately 1 × 10^5^ parasites per mL (p/mL) (denoted with black dotted line, L.O.D.). End of line indicates when mice were removed from the experiment upon reaching endpoint criteria. (*C*) Western blot showing relative APOL1 concentration in plasma of the singular HET that developed parasitemia in *B*. 2 uL of blood taken weekly was diluted 1:20 in IMDM, and diluted serum was frozen for gel loads. 0.5 μL of serum was loaded and probed with anti-APOL1.

The data thus far showed that coexpression of *APOL1* (stable, germline) and *HPR* (HGD, transient) provided better protection than *APOL1* alone (Table 1 and Fig. 4*B*). We next generated mice that stably coexpress *APOL1* and *HPR* in the germline. The *P. hamadryas HPR* cDNA was expressed via an albumin promoter to drive liver-specific expression of the transgene, while *APOL1* was maintained under the same ubiquitin promoter used previously (*SI Appendix*, Fig. S6*A*). These mice produced similar APOL1 levels compared to our previous line of mice, and they produced HPR protein at a concentration that was comparable to *P. hamadryas* (*SI Appendix*, Fig. S6*B*). Heterozygote *APOL1/HPR* mice were fully protected from infection against a *T. b. brucei* strain that can infect mice that only express *APOL1* (*SI Appendix*, Fig. S6*C*). However, the protection against infection by *T. b. rhodesiense* was similar to the mice expressing only *APOL1* (*SI Appendix*, Fig. S6*D*). We also generated mice that coexpressed *APOL1* and *HPR* on separate ubiquitin promoters, anticipating that simultaneous expression of both genes would facilitate protein cosecretion onto HDLs (*SI Appendix*, Fig. S6*E*). These heterozygote mice were fully protected against *T. b. rhodesiense* (Fig. 5*A*). This germline Tg mouse model provides complete protection against *T. b. rhodesiense*. When infected with *T. congolense,* however, we observed the same trend of homozygote mice being protected while some heterozygotes remained susceptible (Fig. 5*B*). In repeat challenges, all mice were protected, meaning only one of 19 mice challenged developed parasitemia (Table 1). To test whether the APOL1 plasma concentration fluctuated in response to infection, serum samples from throughout the infection were probed for APOL1, showing consistent expression (Fig. 5*C*). We therefore conclude that this model still has insufficient concentration of circulating APOL1 to provide complete protection against all relevant trypanosome species.

### APOL1 Chimeras Fully Protect Mice from Trypanosome Infection.

**Fig. 6.**
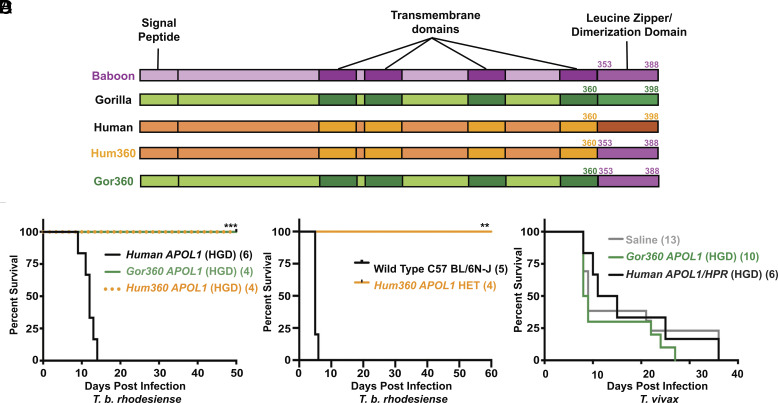
Expression of *APOL1* chimeras fully protects mice from some trypanosome infection, but not *T. vivax*. (*A*) Illustration of the putative domain architecture of APOL1 (determined using JPred4) from human *APOL1*, gorilla *APOL1*, *P. hamadryas APOL1*, and the chimeric *APOL1* genes, which encode the first 360 amino acids of either the human (*Hum360*) or gorilla (*Gor360*) APOL1 protein fused to the 36 C-terminal amino acids of the *P. hamadryas* APOL1 protein, resulting in human/gorilla 4th transmembrane domain and baboon C-terminal leucine zipper motif. (*B*) Kaplan–Meier survival curve of mice expressing chimeric *APOL1* or human *APOL1* by HGD inoculated with 5,000 *T. b. rhodesiense* KETRI243 parasites i.p. (***P = 0.0001; Log-rank test). (*C*) Kaplan–Meier survival curve of WT and Tg HET mice expressing chimeric *APOL1* (*Hum360*) on a ubiquitin promoter, inoculated with 5,000 *T. b. rhodesiense* KETRI243 parasites i.p. (**P = 0.0035; Log-rank test). (*D*) Kaplan–Meier survival curve of Swiss Webster mice expressing chimeric *APOL1* (*Gor360*) or human *APOL1* and *HPR* (ubiquitin promoters) by HGD inoculated with 5,000 *T. vivax* IL1392 parasites i.p. (ns, *P* = 0.4302; Log-rank test). Experiment was repeated with a second isolate, with similar results.

Since coexpressing *HPR* was not sufficiently protective by this genetic modification strategy, we considered increasing the levels of circulating APOL1 to generate fully protected mice. Human *APOL1* expressed in mice by HGD using similar constructs presented here circulates at similar concentrations as APOL1 in humans (43–45), while *P. hamadryas APOL1* expressed in mice (germline) circulates at a slightly lower concentration than in *P. hamadryas* baboons (Fig. 4*A*). Given that both of these models use the same expression system, we reasoned that something intrinsic to the protein coding region of the gene dictates the differences in the amount of protein detectable in plasma. In order to generate mice that produce APOL1 levels high enough to protect from the important veterinary trypanosomes, we decided to produce *APOL1* chimeras encoding the majority of either the human or the closely related gorilla *APOL1* coding sequence fused to the C-terminus of the *P. hamadryas APOL1* (human or gorilla amino acids up to and including residue 360, followed by *P. hamadryas* residues until the C-terminus) (*Hum360* or *Gor360*, Fig. 6*A*). The minimum-required portion of the C-terminus for ideal trypanolytic function was chosen based on the interactions of the C-terminus and the SRA protein (19, 21). The gorilla sequence was synthesized to mitigate any potential future complications related to using human DNA in a genetically modified organism. Both chimeras were expressed at levels equivalent to human APOL1 when expressed in mice by HGD (*SI Appendix*, Fig. S8*B*), and both chimeras fully protected mice from infection by a clinical isolate of *T. b. rhodesiense* (Fig. 6*B*). We moved forward with these constructs and generated mice expressing either the *Hum360 APOL1* or *Gor360 APOL1* as stable Tg animals on ubiquitin promoters. Chimeric APOL1 loaded onto HDLs as expected, with little to no lipid-free APOL1 detectable in plasma (*SI Appendix*, Fig. S7), which is characteristic of human (46) and primate HDL (47). Heterozygote Tg *Hum360* mice were protected against *T. b. rhodesiense* (Fig. 6*C*). However, we have not been able to generate *Hum360* homozygote animals, and *Gor360* males were never able to sire. These experiments have provided the first two *APOL1*-only constructs capable of fully protecting mice from *T. b. rhodesiense* through our genetic modification strategies: the chimeric Hum360 (transient and stable) and the Gor360 (transient only). These constructs illustrate that high APOL1 plasma protein levels are one strategy by which full trypanosome protection can be afforded, provided that toxicity is mitigated.

### Transient Expression of *APOL1* in Mice Does Not Protect from *T. Vivax* Infection.

Using these optimized constructs, we interrogated the long-standing assumption that APOL1 also provides immunity to *T. vivax* (31). The experiments were performed with uncharacterized field isolates of *T. vivax*, so we first confirmed the identity of the parasites by PCR using well-established *T. vivax*-specific primers (*SI Appendix*, Fig. S8*D*). Upon species confirmation, we performed transient expression-based infections in mice producing either human *APOL1* or the gorilla/baboon chimeric variant *Gor360*, and ultimately observed no APOL1-mediated protective effect in either group (Fig. 6*D* and *SI Appendix*, Fig. S8*C*). These data are consistent with studies using human serum administered to mice as a curative therapy, which revealed a general serum-resistance phenotype in *T. vivax* isolates (48). This suggests that APOL1 alone, or in combination with HPR, is insufficient to provide protection against *T. vivax*.

## Discussion

TLFs are primate-specific innate immune complexes that circulate in plasma. The lytic component of TLFs is the APOL1 protein, which forms cation channels in membranes and leads to osmotic swelling of *T. b. brucei* parasites. Here, we reveal that the *Papio spp*. variants of the APOL1 protein are a broadly acting trypanocidal protein that is less restricted by pH for channel formation (7). Further, we find that the *Papio spp*. *APOL1* is polymorphic. Some of these polymorphisms have functional implications in protection against African trypanosomes, in particular the amino acid substitutions that differentiate *P. hamadryas* and *P. cynocephalus* (Figs. 1, 2 *A* and *C*, and 3*E*), and the C-terminal K379 and K380 of *P. hamadryas* APOL1 (36). We have generated several genetically modified mice that express the cDNA of the *P. hamadryas APOL1, or chimeras,* and have created a model system whereby the interaction between APOL1 and other pathogens can be investigated. Since the *APOL1* expression system in our mice is not cell-type specific, our genetically modified mouse model is the best existing system to analyze APOL1–pathogen interactions in a physiological setting, for pathogens that infect mice. As one example, our *APOL1* Tg mice are protected from *Leishmania major* infection (49). *APOL1* expression is upregulated by interferon signaling, suggesting that it also plays an antiviral role, and indeed *APOL1* overexpression was shown to limit HIV replication in vitro (13). We maintain that the study of primate APOL1 antimicrobial function and cell biology must involve the use of physiologically relevant systems, such as those presented here. In this manuscript, we are specifically interested in modeling the feasibility of genetically modified livestock resistant to trypanosome infection.

Before generating the mouse models of *Papio spp.* APOL1 expression, we investigated the trypanolytic mechanism of *Papio* APOL1 in vitro. We empirically show that *P. hamadryas* and *P. cynocephalus* APOL1 ion channel formation are less restricted by pH than human APOL1 (Fig. 3). *P. hamadryas* APOL1 inserts into lipid membranes from pH 5 to 7.2 pH (human APOL1–only acidic), and channels open from pH 5 to 7.2 (human APOL1–only neutral) (Fig. 3*C*). This property of *Papio ssp.* APOL1 could explain the protein’s ability to lyse *T. b. gambiense* parasites (Table 1 and *SI Appendix*, Fig. S2*C* and Table S4), suggesting that APOL1 may insert into the endosomal membrane prior to reaching the TgsGP-containing compartments. However, under physiological conditions, the APOL1 protein is delivered to trypanosomes by a TLF complex. Therefore, to insert in a lipid membrane, the APOL1 likely requires a yet-to-be-characterized TLF-disassociation step that may require a decrease in the environmental pH, with the endolysosomal protease Cathepsin-L, and its potential regulator inhibitor of cysteine peptidase, possibly involved (26). These aspects of the mechanism cannot be studied using recombinant bacterially derived proteins. The Tg mouse models presented allow for the investigation of these mechanisms using human, baboon, or chimeric APOL1 proteins on HDLs as a TLF complex. The relevance of these systems is also supported by the PTMs present on at least the *Papio* variants of APOL1 (*SI Appendix*, Fig. S3). We have illustrated here that the *P. hamadryas* variant is at least N-glycosylated, and a mass-spec based approach could be used in the future to identify additional PTMs present on the protein. The location, size, and type of the PTMs on the *Papio* variants may inform as to the mechanistic detail of membrane insertion and/or channel formation.

APOL1 resistance in *T. congolense* and *T. vivax* has been observed in field isolates (50) and *T. congolense* resistance can be induced by serial passage in the presence of low concentrations of human serum (40). We and others hypothesize that many livestock-infective trypanosomes are capable of developing APOL1 resistance, but these isolates rarely establish human infections with only approximately 20 documented cases of “atypical” trypanosomiasis in the past century, only one caused by *T. congolense* (51, 52). We hypothesize that while TLF mediates primate immunity to *T. b. brucei* and *T. b. evansi* infection, there are likely more factors involved that are responsible for cooperating with TLF to control *T. congolense*, *T. vivax*, *T. lewisi* (53), *T. musculi* (54), and other nonhuman infective, yet seemingly APOL1-resistant, parasites. One possible explanation involves the concentration of TLF in blood versus extravascular spaces. The low-TLF niches may select for the emergence of TLF-resistance in surviving parasites. These parasites, when evolving APOL1 resistance, may have adapted a fitness cost, given the elongated time of emergence of parasitemia when mice were infected with APOL1-resistant *T. congolense* (Fig. 4*D*). These parasites, while TLF resistant, may then be incapable of establishing clinical infections in their primate hosts. Another possibility is that fluctuating serum-APOL1 levels from day to day in a given mouse could temporarily create an environment more suitable for resistance emergence in this experimental system, though we saw that Tg mice with APOL1-resistant parasites had consistent APOL1 expression (Fig. 5*C*). More generally, it is possible that TLF resistance in *T. congolense* and *T. vivax* is not sufficient to mediate primate infectivity. Many of these hypotheses could be addressed in the future by performing in vivo baboon challenges with Tg murine generated TLF-resistant strains. These or similar studies seem particularly relevant given the maintained assumption that human immunity to *T. vivax* is mediated by APOL1 (31). The data presented here challenges that assumption, suggesting the mechanism of primate immunity to *T. vivax* and *T. congolense* is more complex than is currently understood. Further, given how readily *T. congolense* APOL1 resistance is established in our mouse models (Table 1), we hypothesize that this species may inherently harbor APOL1 resistance mechanisms, as opposed to a series of different de novo mutations occurring in each of the HET mice that eventually displayed a parasitemia. These mechanisms, when activated/upregulated, could permit survival in the presence of APOL1 similarly to what occurs in *T. b. rhodesiense* and *T. b. gambiense*, although perhaps these *T. congolense* mechanisms are typically too toxic to the parasites to become prevalent in the field, given that this parasite does not commonly infect humans. Still, it would be prudent to identify the genetic elements within *T. congolense* that allow for APOL1 resistance; we hypothesize that at least some of them may be housed within variant surface glycoprotein (VSG) expression sites similarly to the *T. b. rhodesiense SRA* gene, and that a combination mechanism may be necessary for survival and proliferation [which is also the case for *T. b. gambiense* (55)]. This hypothesis is supported by the time until parasitemia onset; even assuming that only one resistant parasite survives from the initial inoculum, mathematically one would still expect parasitemia to reach detectable levels much sooner than 30 d postinfection, and these low-inoculum experiments have already been done in mice with *T. b. brucei* (56). Instead, we hypothesize that a subpopulation of parasites already expressing APOL1 resistance is/are capable of quiescently surviving in extravascular APOL1-low niches in the body until eventually a VSG expression site switch occurs, leading to the expression of the remaining resistance factors. However, there remains debate with respect to whether data obtained from *T. vivax* experiments performed in mouse models can be clearly interpreted. Given that the majority of *T. vivax* isolates cannot be cultivated in mice, this questions the relevance of those isolates that can infect mice (31).

In order to create animal models that are protected against trypanosome infections as well as primates are, it stands to reason that the APOL1 and HPR proteins would need to colocalize onto the same HDL particles, thus recreating TLFs. So far, it has proven difficult to achieve complete protection in Tg mice using this strategy (Fig. 5 and *SI Appendix*, Fig. S6). We are unsure of the cause; there may be other HDL synthesis mechanisms or proteins that play roles in primate TLF assembly that have not yet been characterized and do not exist in mice. TLF assembly has generally not been extensively investigated, although we suggest that expanding this literature may prove useful in the contexts of APOL1-related immunology as well as APOL1-related tissue toxicity. However, a final challenge is that these Tg murine models do not produce TLF2, the lipid-poor IgM-containing TLF complex. TLF2 facilitates a secondary route of APOL1 uptake by trypanosomes, possibly through IgM–VSG interaction and some have proposed it to be the predominant killing complex in human serum (57). We have recreated TLF2 in mice lacking activation-induced cytidine deaminase and thereby producing higher-than-WT levels of natural IgM antibodies (57). This would not be a good strategy in livestock, therefore, we did not pursue this line of enquiry.

Nevertheless, a second strategy for generating fully protected animal models is to increase APOL1 protein expression level (Fig. 6). This was achieved by making chimeric APOL1 proteins. It remains unclear why there is less APOL1 in *Papio* plasma than the APOL1 in human plasma (Fig. 2*B*). Given that the Tg murine models presented here used the same expression system with either *P. hamadryas* or chimeric human/baboon protein, and had the same levels as the baboon plasma and human plasma respectively (Fig. 6 and *SI Appendix*, Fig. S6*B*), we hypothesize that this differential concentration is not based on RNA expression. We instead hypothesize that the *P. hamadryas* APOL1 protein is not as efficiently secreted and loaded onto HDLs, or that *P. hamadryas* APOL1 is degraded by serum proteases more efficiently than human APOL1 after secretion. The C-terminal region of *P. hamadryas* APOL1, which is necessary and sufficient for *T. b. rhodesiense* lysis, does not apparently decrease the efficiency of HDL loading or protein secretion as evidenced by the chimeric APOL1 proteins created here (Fig. 6). However, these chimeric proteins were evidently produced too efficiently, given the associated developmental fitness cost because we could not breed homozygote mice. We believe that the chimeras are strong candidate genes to be used in future murine and livestock studies after titrating their expression levels with slightly weaker promoters.

Our long-term research goal is to investigate the possibility of creating trypanosome-resistant livestock that could thrive in the tsetse endemic regions of sub-Saharan Africa through Tg expression of *APOL1*. To this end, we have used somatic cell nuclear transfer to produce a cloned Kenyan Boran bull in Africa as critical first step (58). Trypanosome-resistant cattle breeds could expedite agricultural development and significantly reduce the rates of human trypanosomiasis transmission in sub-Saharan Africa (59), especially when combining their impact with additional control efforts. In addition, we hosted two stakeholder meetings at the International Livestock Research Institute in Nairobi Kenya to discuss the impact of Tg livestock in Africa (60, 61).

The use of APOL1-Tg cattle as an intervention strategy requires the complete protection of the animal against all livestock trypanosomes (*T. congolense, T. vivax*) without adverse effects on the health of the animal. Our mouse models showed that the use of *P. hamadryas APOL1* with *HPR* provided complete protection against human-infective *T. b. rhodesiense*, which could translate to a reduction in the reservoir for human disease. However, these mice were ultimately not completely protected against *T. congolense*, with the low concentration of APOL1 selecting for resistant parasites (Fig. 4*B*). These resistant parasites remained resistant at higher concentrations of APOL1, increasing their virulence (Fig. 4*C*). In the field, this could actually facilitate an increase in human-infective trypanosome species and reservoirs, making the project too risky of an endeavor. Mice expressing substantially higher levels of chimeric APOL1s offer a promising route forward, albeit with an apparently deleterious developmental effect in the context of the mice presented here (Fig. 6*C*). The promoter could be modulated to fine-tune *APOL1* expression to a level that is completely protective without any toxicity impact, although this “Goldie Locks” level of expression may be different between mice and cattle, posing additional developmental challenges. Regardless, our preliminary *T. vivax* data suggest that APOL1 (*P. hamadryas* or *H. sapiens*) does not protect against this parasite (Fig. 6*D*). If this observation is validated using additional experiments, and given that *T. vivax* is distributed throughout South America, Africa, and Asia, then *P. hamadryas APOL1* Tg cattle will ultimately prove to be ineffective. Additionally, the seemingly common emergence of APOL1 resistance in these murine models underscores the need for continued efforts to improve our understanding of APOL1–parasite interactions prior to initiating any actual Tg livestock studies. Clearly, sublethal levels of APOL1 can select for the emergence of resistance in vivo, which bears relevance in Africa, where human-infective species are endemic, to the ongoing APOL1-expression-reducing (62) and channel blocking (63) clinical trials in the nephrology space. Indeed, we hope that the biology presented here will assist us and others in our efforts to illuminate the evolutionary relationship between primate *APOL1* and trypanosomes.

## Methods

Parasite strains, antibodies, and primate samples are described in the *SI Appendix*.

### Phylogenetic Analysis of Papio *APOL1*.

The genotype data generated originally by the Baboon Genome Project were downloaded at ftp://ftp.hgsc.bcm.edu/Baboon/Panu_2.0/ which is contributed by Baylor College of Medicine Human Genome Sequencing Center. The dataset consists of 16 individuals belonging to six species within the genus *Papio*, and *T. gelada*, a member of a closely related genus that serves as an outgroup (30). Details found in *SI Appendix*.

### Production and Purification of rAPOL1s.

We expressed and purified N-terminally 6xHIS-tagged full-length rAPOL1 using the pNIC vector from *E. coli* BL21 Codon Plus RIPL cells (Agilent) grown in Overnight Express media (Novagen) using a previously described procedure with slight modification (7, 9). Details found in *SI Appendix*.

### Western Blotting.

All western blots were performed using polyvinylidene difluoride membranes after overnight electro-transfer at 30 mV. Membranes were blocked with 5% milk powder in 150 mM NaCl, 50 mM Tris, pH 7.5. Membranes were probed with both primary and secondary antibodies diluted in the blocking buffer with interspaced washes (3 × 10 min) with 150 mM NaCl, 50 mM Tris, pH 7.5 prior to chemiluminescent visualization (Thermo Scientific). All washes and probes were conducted in the presence of 0.05% Tween-20 (Fisher Scientific). For all serum based western blots, loading was controlled by volumetric measurements.

### Trypanosome In Vitro Lysis Assays.

Cultured parasites were diluted to 5 × 10^5^ cells/mL in HMI-9 and added to a 96-well plate. Parasites were diluted 1:1 with various concentrations of rAPOL1 proteins suspended in HMI-9. AlamarBlue (Invitrogen) was used to quantify nonlysed cells. Details found in *SI Appendix*.

### In Vivo Parasite Infections.

All experiments conducted in mice were approved by the Institutional Animal Care and Use Committees of the appropriate institutions. For trypanosome infections, 5,000 parasites (unless otherwise indicated) were injected intraperitoneally (i.p.) into Tg or WT C57BL/6N-J mice derived from founder mice expressing *P. hamadryas APOL1*, *P. hamadryas HPR*, and/or human APOA-I. Parasitemia was monitored by tail bleeding. Mice that were transiently transfected with any HGD construct were infected 48 h after plasmid injection. Details found in *SI Appendix*.

### Lipoprotein Isolations.

The density of *P. hamadryas* or mouse plasma was adjusted to 1.25 g/mL using KBr and samples were ultracentrifuged in an NVTi-65 rotor (Beckman Coulter) for 16 h at 49,000 RPM, 10 °C. The top 33% of the resulting gradient was aspirated and dialyzed against five changes of 150 mM NaCl, 50 mM Tris, 0.5 mM Ethylenediaminetetraacetic acid (EDTA), pH 7.5 before storing at −80 °C.

### Deglycosylation of *P. Hamadryas* Lipoproteins.

PNGase F was obtained from New England BioLabs and deglycosylation was performed in 50 μL under denaturing conditions according to the manufacturer’s instructions except that 1 mM EDTA, 1 mM EGTA, 1 mM AEBSF (4-(2-Aminoethyl)-benzenesulfonyl fluoride hydrochloride) (final concentrations), and 0.33 μL of the HALT protease inhibitor cocktail (Thermo Scientific) were added to the lipoproteins prior to denaturation.

### Generation of Transiently Tg Mice.

Expression of *P. hamadryas HPR*, or chimeric *APOL1s*, was achieved using HGD (18). Briefly, mice were injected through the tail vein with 10% of their body weight in sterile 0.9% NaCl solution containing 25 μg of plasmid DNA encoding the cDNA of the gene of interest flanked by an SV40 polyadenylation sequence and a beta-globin intron under the control of a human ubiquitin promoter. Prior to trypanosome infections, blood was drawn from the tail 2 d posttransfection to confirm protein expression by western blot (data from all HGD experiments is compiled in *SI Appendix*, Fig. S8).

### Histology.

Kidneys were excised from >1-y-old mice. The kidneys were sliced into two longitudinal halves and washed in phosphate-buffered saline before being fixed overnight in 4% paraformaldehyde at 4 °C. The kidneys were then transferred to 70% ethanol in phosphate-buffered saline for storage. The kidneys were submitted to the New York University pathology core facility. 5 μm sections were cut, fixed in formalin, and embedded in paraffin prior to being stained with hematoxylin and eosin.

### *T. Congolense* qPCR.

To design primers for quantitative PCR specific to *T. congolense* parasites, we targeted the *T. congolense CATL* gene. *CATL* is a multicopy gene, existing in up to 20 copies in some isolates, making it an ideal candidate gene for highly sensitive PCRs. Using previously published broadly specific *CATL* primers, we amplified and sequenced the gene from *T. congolense* STIB-68-Q. We then designed primers (F: 5’-GATCTTCGCACCAACGACCT, R: 5’- AAGGGCAATGTGTTCACGGA) that amplified a 90 bp segment of the gene for quantitative PCR. The quantitative PCR cycling conditions are as follows: Initial denaturation at 94 °C for 1 min, 38 cycles of 98 °C for 5 s, 59.5 °C for 30 s, and 72 °C for 20 s. PCRs were performed in 20 μL final volumes consisting of 10 μL of SYBR select Master Mix (Applied Biosystems), and 10 μL of remaining components amounting to 30 ng of template DNA and 0.5 μm primer concentrations.

### Tissue Collection for qPCR.

Infected mice were manually transcardially perfused through the left ventricle after aspirating the right atrium with 20 mL of ice-cold heparinized phosphate-buffered saline. After cutting the atrium and prior to starting the injection of saline, 100 μL of blood was collected from the thoracic cavity and immediately pipetted into 500 μL of TriReagent (Zymo Research). After perfusing the animal, sections of the lung, liver, gonadal adipose depot, spleen, and brain were excised, washed in phosphate-buffered saline to remove residual blood, weighed, and placed into 500 μL of TriReagent (Zymo Research). Tissue samples were stored at 4 °C until homogenization by sequential needle passing. Tissue homogenates were then mixed with an equal volume of molecular grade ethanol before isolating genomic DNA using a commercially available kit (DNeasy Blood and Tissue Kit - Qiagen).

### Electrophysiology.

Planar lipid bilayers were formed at room temperature from soybean asolectin and cholesterol (Sigma, C8667) across an 80- to 120-μm hole in a Teflon partition separating two solutions of bilayer buffer (1 mL vol), as described previously (9). Bilayer buffer: 150 mM KCl, 5 mM CaCl_2,_ 0.5 mM EDTA, 5 mM K-succinate, 5 mM K-HEPES, pH 5.5-7.5. Details found in *SI Appendix*.

### Generation of Chimeric *APOL1* Genes.

The “*Hum360*” chimera was obtained from Thomson and Genovese et al. (15, 21). To generate the “*Gor360*” chimera, the gorilla (*Gorilla gorilla gorilla*) *APOL1* gene was assembled through PCR of each individual *APOL1* exon from gorilla genomic DNA obtained from the Wildlife Conservation Society in Bronx, NY. The exon flanking primers were designed using an assembled gorilla genome (gorGor_Susie3) obtained from the Eichler Lab at the University of Washington European Nucleotide Archive, Project Accession PRJEB10880, Taxonomy_ID: 9595 (64). Primers used found in *SI Appendix*. The sequenced exons were stitched together manually in silico, and the full-length gene was then synthesized by Invitrogen GeneArt Gene Synthesis Service by Thermo Fisher Scientific. The “*Gor360*” chimera was then created through In-Fusion cloning (Takara) by fusing the first 1,080 bp of the gorilla gene with the C-terminal region of *P. hamadryas APOL1* (accession: FJ429176). All PCRs were performed using Pfu Ultra AD high-fidelity polymerase (Agilent Technologies).

### Generation of Targeted *P. Hamadryas APOL1*-Expressing Mice.

For targeting constructs containing the *P. anubis APOL1* genomic sequence, all sequences were isolated from a BAC obtained from the Children’s Hospital Oakland Research Institute. All genomic coordinates are from the Panu_3.0/papAnu4 version of the *P. anubis* genome in the UCSC genome browser. The methods for generating the targeting constructs found in *SI Appendix*. All constructs were electroporated into either C57BL/6NTac or hybrid C57BL/6NTac:129S6/SvEvTac embryonic stem cells (ESC). Loss- and gain-of-allele PCR analyses were used to confirm genome modifications in ESC clones and genotype live mice, as described in Valenzuela et al. (65). All assay sequences are listed in *SI Appendix*, Table S5. All antibiotic selection cassettes were removed using either a Cre recombinase expression vector in the targeted ESC clone or during male germline maturation. HET targeted cells were microinjected into 8-cell embryos from Swiss Webster albino mice (Charles River Laboratories), yielding F0 VelociMice that were 100% derived from the targeted cells (C57BL/6NTac or hybrid C57BL/6NTac:129S6/SvEvTac) (66). These mice were subsequently bred and maintained in the Hunter College Animal Facility during the study period. All relevant protocols were approved by the corresponding Institutional Animal Care and Use Committees of Regeneron and Hunter College.

## Supplementary Material

Appendix 01 (PDF)

## Data Availability

Data cannot be shared. Regeneron materials described in this manuscript may be available to qualified, academic, noncommercial researchers upon request through our portal (https://regeneron.envisionpharma.com/ienv_research/visiontracker/portal/login.xhtml?pgm=ISR&windowId=4d6). Regeneron does not share clinical molecules. Regeneron does share alternative molecules that behave in a similar manner. For any questions about how Regeneron shares materials please connect with Regeneron using the preclinical collaborations email address (preclinical.collaborations@regeneron.com). Previously published data were used for this work (PRJNA260523) (30).
