# Supplementary material for: Germline-targeted baboon apolipoprotein L-1 protects mice against African trypanosomes
Source: Proc Natl Acad Sci U S A. 2026 Mar 27;123(13):e2525773123. doi: 10.1073/pnas.2525773123 (PMC13037889; doi:10.1073/pnas.2525773123)
Supplement: Supplementary file 1 — Appendix 01 (PDF) [file pnas.2525773123.sapp.pdf]

Supporting Information for
Germline Targeted Baboon Apolipoprotein L-1 Protects Mice Against African Trypanosomes

Sara Fresard[1, 2], Sarah J. Pangburn[1, 2], Kayla Leiss[2], Daphne Boodwa-Ko[2], Daniella Kovacsics[2], Chris J. Schoenherr[3], Jeremy S. Rabinowitz[3], Aris N. Economides[3], Li Li[1,2], Weigang Qiu[1,2], Bernardo Gonzalez-Baradat[2], Alessandro Rosa[1, 2], Russell Thomson[2], Jayne Raper[1,2*] and Joseph Verdi[1,2*]

1 Biology Program, The Graduate Center at the City University of New York, New York City, NY 10016 USA

2 Department of Biological Sciences, Hunter College at the City University of New York, New York City, NY, 10065 USA

3 Regeneron Pharmaceuticals, Tarrytown, NY, 10591 USA

Corresponding Author: Joseph Verdi, j.verdi@dkfz-heidelberg.de

This PDF file includes:

Detailed methods
Figures S1 to S9
Tables S1 to S5
SI References

SI Text

Detailed methods

Trypanosome strains. *T. b. brucei* Lister 427 and *T. b. gambiense* ELIANE were grown *in vitro* in HMI-9 with 10% fetal bovine serum and 10% Serum Plus at 37°C in 5% CO_2. *T. b. brucei* Lister 427-SRA expresses the SRA gene from *T. b. rhodesiense,* generated by Cross lab. The other trypanosomes were maintained exclusively in mice: *T. b. rhodesiense* KETRI243, *T. b. brucei* Antat1.1, *T. b. evansi* Antat3, *T. vivax* IL1392, and *T. congolense* STIB-68-Q.

***T. vivax* species confirmation**. A parasite isolate characterized as *T. vivax* was experimentally inoculated in mice at the International Livestock Research Institute. To confirm that the isolate was *T. vivax*, DNA was isolated from infected mouse blood or uninfected mouse blood as a negative control. DNA was amplified using established thermocycling conditions and *T. vivax*-specific primers[1]. Forward: GCCATCGCCAAGTACCTCGCCGA Reverse: TTAAAGCTTCCACGAGTTCTTGATGATCCAGTA

Primate samples. Primate plasma samples were obtained from the North Carolina Zoological Park, Asheboro, NC.

Antibodies. For the detection of baboon APOL1, we generated a novel antibody through AnaSpec Incorporated, which was raised in rabbits against the following peptide: CSVEERARVVEMERVAESRTTEVIRGAKIVDK. An anti-human APOL1 antibody (ProteinTech, 11486-2-AP) was used for the detection of human, gorilla, or chimeric APOL1. The anti-human HP that recognizes *P. hamadryas* HPR is commercially available (Sigma, H8636). The secondary antibody used in all western blots was the anti-rabbit TrueBlot conjugated to HRP (1:5000) (Rockland Antibodies, 18-8816-33).

Phylogenetic analysis of Papio *APOL1*. The genotype data generated originally generated by the Baboon Genome Project were downloaded at ftp://ftp.hgsc.bcm.edu/Baboon/Panu_2.0/ which is contributed by Baylor College of Medicine Human Genome Sequencing Center (HGSC). The data set consists of 16 individuals belonging to six species within the genus *Papio*, and *Theropithecus gelada*, a member of a closely related genus that serves as an outgroup[2]. We extracted variant calls in *APOL1* exons by filtering nucleotide position using VCFtools (version 0.1.17)[3], where we retrieved 39 single nucleotide polymorphism (SNP) sites and 3 indels. The variant sites of the 32 samples were aligned using a customized PERL script and a maximum likelihood tree of the haplotypes was inferred with FastTree (version 2.1.8)[4]. The haplotypes and the tree were then visualized in Adobe Illustrator 2018. To estimate nonsynonymous (dN) and synonymous (dS) substitution rates and thereby detect adaptive selection on *APOL1* gene, we reconstituted the full-length codon sequences (1167 nt) of the 32 haplotypes according to a reference sequence. The reference sequence, consisting of 1221 nucleotides, is the mRNA of *APOL1* in *Papio anubis* (NCBI accession NM_001302101). We matched the SNP sites to the reference sequence position by position using a customized pipeline. The dN/dS ratio was then estimated using program PAML V4.9[5] with a neutral model (M0), a nearly neutral model (M1a) and a positive selection model (M2a). Likelihood ratio tests were carried out between models. Typically, lower dN/dS values (<1) indicate negative selection is occurring, while higher values (>1) indicate positive selection is occurring. PAML output also includes a rate ratio (tS/tV) of transitions (purine to purine or pyrimidine to pyrimidine substitutions) to transversions (purine to pyrimidine or pyrimidine to purine substitutions). A higher tS/tV value indicates a higher rate of transitions, which has been associated with mutational constraint, while a lower value suggests that transitions and transversions are equally well tolerated and has been associated with more rapid evolution[6]. However, the relevance of protein/function-based selection to the tS/tV ratio confounded by additional factors[7], making dN/dS the more robust statistic between the two.

Production and purification of rAPOL1s. We expressed and purified N-terminally 6xHIS-tagged full length rAPOL1 using the pNIC vector from *E. coli* BL21 Codon Plus RIPL cells (Agilent) grown in Overnight Express media (Novagen) using a previously described procedure with slight modification[8,9]. Inclusion bodies were washed in the presence of the protease inhibitors AEBSF and EDTA, then solubilized in 1% zwittergent 3-14 (SB3-14, EMD-Millipore). Solubilization was facilitated by briefly adjusting to pH 12 with 10 mM NaOH and 150 mM NaCl for one minute before adding 30 mM Tris-HCl (pH 7.4) to reduce the pH to 8.0. APOL1 proteins were then separated on a

Superdex-200 16/60 size-exclusion column (GE Life Sciences) equilibrated in 50 mM Tris-HCl, pH 8.5, 150 mM NaCl, and 0.5% SB3-14. APOL1-containing fractions were pooled and bound to a nickel affinity column (HisTRAP, GE Life Sciences) equilibrated in 20 mM Tris-HCl, pH 8.5, 150 mM NaCl, 10 mM imidazole and 0.5% SB3-14 and eluted with a 10-500 mM imidazole gradient in the same buffer. APOL1 proteins were purified from any remaining detectable contaminants via a second size-exclusion protocol using the same size-exclusion column equilibrated in 50 mM Tris-HCl, pH 8.5, 150 mM NaCl, and 0.05% n-Dodecyl-beta-Maltoside (DDM) (Thermo Scientific). Purified proteins were flash-frozen and stored at -80°C.

Trypanosome *in vitro* lysis assays. Cultured parasites were diluted to 5 x 10^5 cells/ml in culture media as described above and 100 µL was added to each well of an opaque 96 well plate. Parasites were then diluted 1:1 with various concentrations of rAPOL1 proteins suspended in HMI-9. The concentration of DDM in the assay was maintained below the theoretical critical micelle concentration (0.006%) and did not affect parasite viability. After 20 hours of incubation at 37°C with 5% CO_2, 20 µL of alamarBlue (Invitrogen) was added to each well. The assay involves the reduction of resazurin by metabolically active cells to the fluorescent resorufin, allowing quantitation of non-lysed cells at 4 hours post-reagent addition in a spectrofluorometer[10].

***In vivo* parasite infections.** All experiments conducted in mice were approved by the IACUC committees of the appropriate institutions. For trypanosome infections, a total of 5000 parasites (unless otherwise indicated) was injected intraperitoneally (i.p.) into transgenic or wild type C57BL/6N-J mice derived from founder mice expressing *P. hamadryas APOL1*, *P. hamadryas HPR*, and/or human APOA-I. Genotype was confirmed by PCR to determine heterozygote vs homozygote status of each individual transgene. Parasitemia was monitored by tail bleeding. Mice were euthanized by isoflurane when parasitemia reached 1 x 10^9 parasites/mL. Mice that were transiently transfected with any HGD construct were infected 48 hours after plasmid injection. All survival experiments were analyzed statistically using the log-rank test via GraphPad Prism. The *T. vivax* infections were performed at the International Livestock Research Institute in Nairobi, Kenya. These studies used Swiss Webster mice and were repeated with two different *T. vivax* isolates with similar results.

Electrophysiology. Planar lipid bilayers were formed at room temperature from soybean asolectin [lecithin type IIS (Sigma Chemical) from which nonpolar lipids had been removed] and cholesterol (Sigma, C8667) across an 80- to 120-µm hole in a Teflon partition separating two solutions of bilayer buffer (1 mL vol), as described previously[8,9]. Bilayer buffer: 150 mM KCl, 5 mM $CaCl_2$, 0.5 mM EDTA, 5 mM K-succinate, 5 mM K-HEPES, pH 5.5-7.5. Briefly, 20 µL of lipid (1.5% asolectin 0.5% cholesterol) in pentane was layered on top of the solutions and the pentane was allowed to evaporate. Bilayers were formed by raising each solution above the hole that was pretreated with squalene. Bilayer formation was monitored by measuring a change in capacitance. Voltages were maintained using the BC-535C bilayer clamp (Warner Instruments) and are given as the voltage of the *cis* solution (defined as the side to which protein was added) with respect to the *trans* solution. The current response was filtered at 30 Hz by a low-pass eight-pole Bessel filter (Warner Instruments) and recorded using IGOR NIDAQ Tools MX 1.0 and IGOR software (WaveMetrics) via an analog-to-digital converter (NI USB-6211; National Instruments). 0.5M HCl (acid) and 0.5M KOH (base) are used to adjust the pH of the *cis* and *trans* sides.

Generation of chimeric *APOL1* genes. The "*Hum360*" chimera was obtained from Thomson and Genovese *et al.*[11,12] To generate the "*Gor360*" chimera, the gorilla (*Gorilla gorilla gorilla*) *APOL1* gene was assembled through PCR of each individual *APOL1* exon from gorilla genomic DNA obtained from the Wildlife Conservation Society in Bronx, NY. The exon flanking primers were designed using an assembled gorilla genome (gorGor_Susie3) obtained from the Eichler Lab at the University of Washington European Nucleotide Archive, Project Accession PRJEB10880, Taxonomy_ID: 9595[13]. Exon 1 F: TGGTCATGGAGGTCAGGATATCGAG; Exon 1 R: CTAGAAGAAGCCCAGATGGCCC. Exons 2 and 3 were sequenced simultaneously because the intron separating the two sequences is relatively small (182 bp). Exons 2 and 3 F: CAGGCCCTGGTCATTGTCAG; Exons 2 and 3 R: CTTGGGGCAGACTCATTGGC. Exon 4 F: GGCTGTTATGCACTCCCAC; Exon 4 R: CTGCCTGGAGGAGGTGTG. Exon 5, the largest exon, was sequenced via a nested PCR. Exon 5 nest F: CTGCCTGGAGGAGGTGTG; Exon 5 nest R: GCATTTTGTCCTGGCCCCG. Exon 5 inner F: GCATTTCCTCTGGCATCCTGAC; Exon 5 inner R: CACGGAGCCTTCTTATGTTA. The sequenced exons were stitched together manually *in silico*, and the full-length gene was then synthesized by

Invitrogen GeneArt Gene Synthesis Service by Thermo Fisher Scientific. The "*Gor360*" chimera was then created through In-Fusion cloning (Takara) by fusing the first 1080 bp of the gorilla gene with the C-terminal region of *P. hamadryas APOL1* (accession: FJ429176). All PCRs were performed using Pfu Ultra AD high-fidelity polymerase (Agilent Technologies).

Generation of targeted *P. hamadryas APOL1*-expressing mice. For targeting constructs containing the *P. anubis APOL1* genomic sequence, all sequences were isolated from a BAC obtained from the Children's Hospital Oakland Research Institute. All genomic coordinates are from the Panu_3.0/papAnu4 version of the *P. anubis* genome in the UCSC genome browser. First, a floxed neomycin expression cassette was inserted into the first intron of *APOL1*, which removed 40bp from the intron (chr10:77,080,500-540). The modified *APOL1* gene (chr10:77,075,579-77,097,083) containing the neomycin (neo) selection cassette was transferred to a R6K origin of replication plasmid. For the *ROSA26* targeting vector, a splice acceptor and polyadenylation sequence from the rabbit beta globin gene was inserted upstream of the *APOL1* promoter to reduce readthrough transcription from the *ROSA26* locus promoter. The two targeting vectors were then constructed by using homologous recombination in *E. coli* to insert the transgene sequences from the R6K constructs into BAC vectors containing either mouse genomic sequences from the *ROSA26* safe harbor or the *Myh9* loci, as described[14]. The *P. anubis APOL1-Myh9* construct was converted to *P. hamadryas APOL1* using homologous recombination to insert the necessary point mutations.

For the UBC-*APOL1* construct, the vector was first assembled in an R6K plasmid and consists of the human ubiquitin C (UBC) promoter and the rabbit beta globin intron upstream of a *P. hamadryas APOL1* cDNA, followed by the bovine growth hormone polyadenylation sequence. For selection in embryonic stem cells, a self-deleting neomycin resistance cassette was placed downstream of the polyadenylation site. For the Alb-*HPR*-UBC-*APOL1* construct, a fusion of the mouse albumin enhancer and promoter sequences[15] and a *P. hamadryas HPR* cDNA with a beta globin polyadenylation sequence was inserted upstream of the UBC promoter in the UBC-*APOL1* construct. For the UBC-*APOL1*-UBC-*HPR* construct, a UBC-driven *HPR* was inserted downstream of the UBC-driven *APOL1* cDNA, and both cDNAs used an SV40 polyadenylation sequence. Targeting vectors were constructed by using homologous recombination in *E. coli* to insert the transgene sequences from the R6K constructs into BAC vectors containing mouse genomic sequences from the *ROSA26* locus, as described above.

Constructs for the chimeric *APOL1* mice (*Hum360 APOL1* and *Gor360 APOL1*) were fully synthesized (GenScript) in an R6K vector. Synthesized DNA included the UBC promoter, the rabbit beta globin intron, the Hum360 or the Gor360 chimeric *APOL1* coding sequence, SV40 polyadenylation sequence and followed by a self-deleting neo resistance cassette. Bacterial homologous recombination was used to insert the construct into a BAC vector for targeting the *ROSA26* locus. All vector sequences are available upon request.

All constructs were electroporated into either C57BL/6NTac or hybrid C57BL/6NTac:129S6/SvEvTac embryonic stem cells (ESC). Loss- and gain-of-allele PCR analyses were used to confirm genome modifications in ESC clones and genotype live mice, as described in Valenzuela et al. 2003[14]. All assay sequences are listed in Supplementary Table 5. All antibiotic selection cassettes were removed using either a Cre recombinase expression vector in the targeted ESC clone or during male germline maturation. Heterozygous targeted cells were microinjected into 8-cell embryos from Swiss Webster albino mice (Charles River Laboratories), yielding F0 VelociMice that were 100% derived from the targeted cells (C57BL/6NTac or hybrid C57BL/6NTac:129S6/SvEvTac)[16]. These mice were subsequently bred to homozygosity and maintained in the Hunter College Animal Facility during the study period. All relevant protocols were approved by the corresponding Institutional Animal Care and Use Committees of Regeneron and Hunter College.

Figures

Supplementary Figure 1. Phylogenetic analysis of the baboon *APOL1* gene.

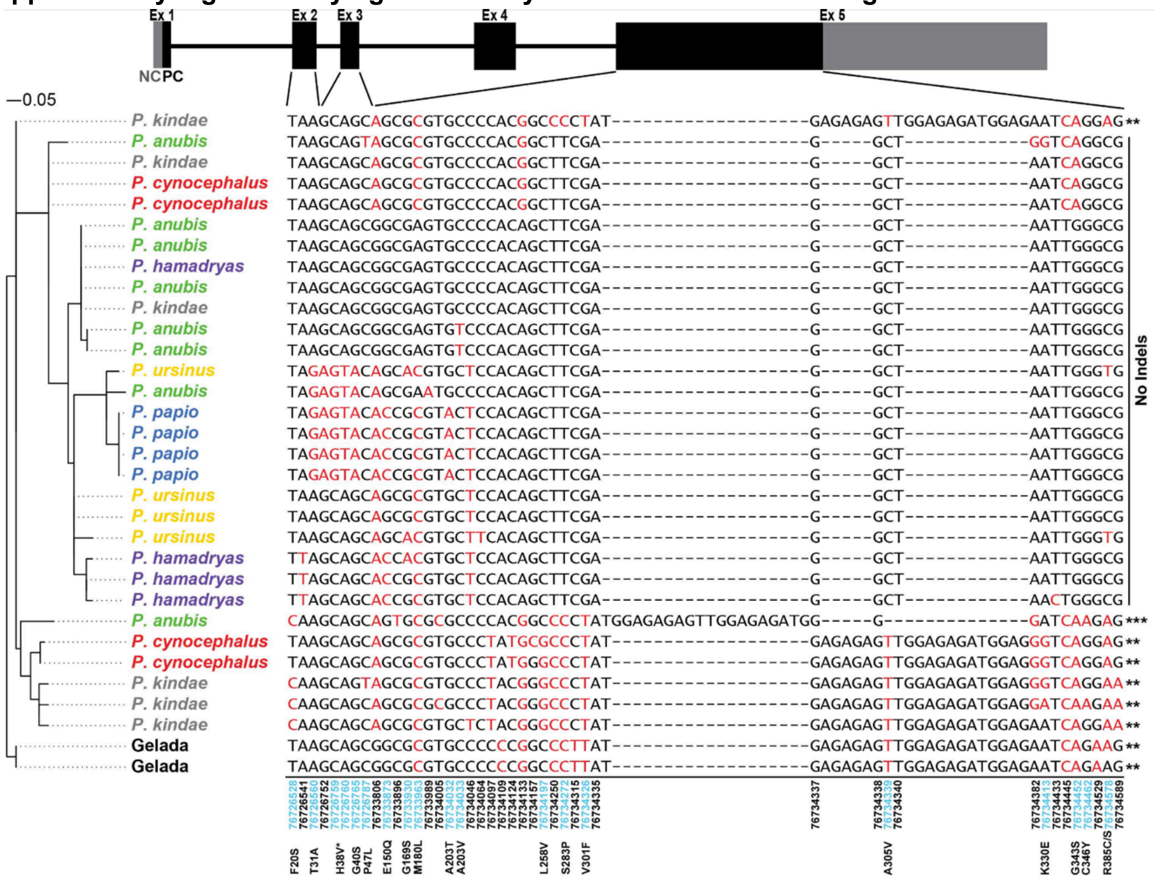

Pictured above the phylogenetic tree and haplotype table is a schematic of the five protein-coding exons of *APOL1* in primates (NC, non-coding; PC, protein-coding). All of the observed polymorphisms were present in exons 2, 3, and 5. Only the sites that are polymorphic between the baboon individuals are shown. Each observed haplotype is depicted separately, with haplotypes grouped by their phylogeny (left). Polymorphisms that do not match the reference sequence (Panu2.0) are red, while reference-matching sites are black. The genetic coordinates of each site in the reference sequence are listed below the table, and polymorphic sites that encode missense mutations (relative to the reference sequence Panu 2.0) are highlighted by the encoded amino acid change above the table. H38V (*) is intentionally located between two SNPs because the amino acid change is encoded by two adjacent polymorphisms. The 'No Indels' and asterisks at the right refer to Supplementary Table 2, wherein the amino acid changes, starting at amino acid position 300, encoded by the different Indel patterns are clarified.

Supplementary Figure 2. Purification of baboon APOL1 made recombinantly in bacteria.

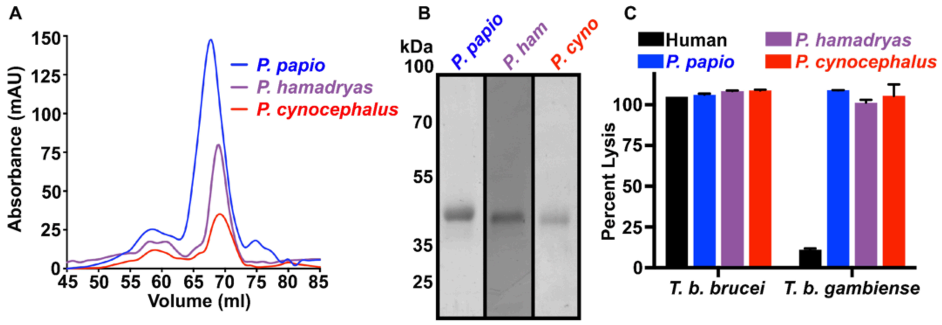

(A) UV absorbance chromatograms of the final size exclusion chromatography step of three different baboon recombinant APOL1 (rAPOL1) protein purifications. (B) Coomassie stained gels of the purified *Papio* rAPOL1 proteins. Expected size: 42 kDa (C) 24-hour trypanolysis assay showing the lytic capacity of 1,250 ng/ml of each of the indicated rAPOL1 proteins against *T. b. brucei* and *T. b. gambiense*. Reproducible data from multiple assays were combined to generate this graph (error bars represent mean +/- SD of three experimental replicates).

Supplementary Figure 3. Human and *P. hamadryas* HPR and APOL1 proteins are post-translationally modified.

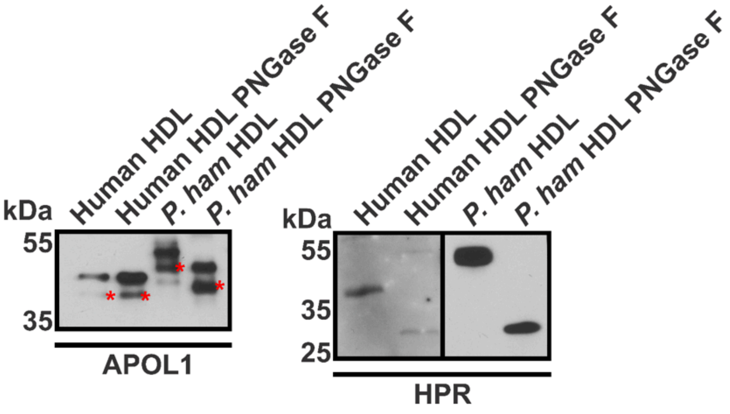

Total HDL lipoproteins were isolated from human and *P. hamadryas* plasma. The HDLs were either treated with vehicle control or PNGase F before electrophoresis under reducing conditions. The molecular mass of *P. hamadryas*APOL1 and both human and *P. hamadryas* HPR decrease significantly after PNGase F treatment, indicating that the proteins are *N*-glycosylated. The red asterisk in the left panel highlights an APOL1 C-terminal cleavage product that often accumulates during sample collection.

Supplementary Figure 4. Integration of the genomic *P. anubis* or *P. hamadryas APOL1* locus in mice is not sufficient to protect from *T. b. brucei*.

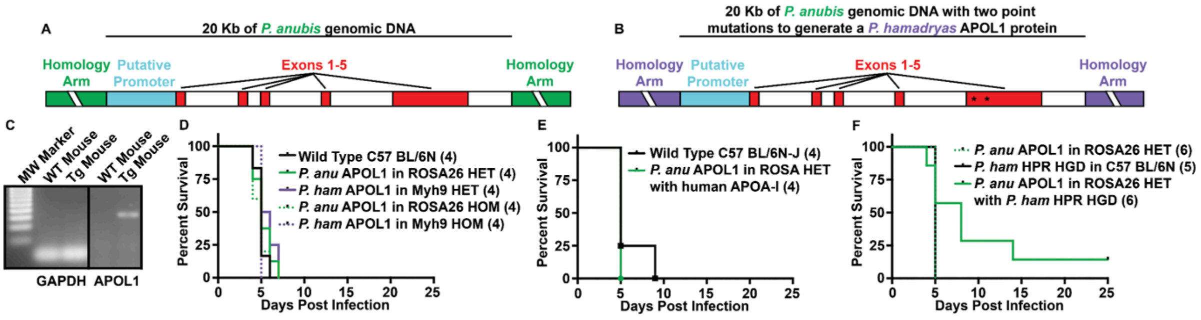

(A-B) Schematics of the targeting constructs used to insert the genomic baboon *APOL1* locus into the mouse genome. The construct in panel B was generated by making two substitutions (*) in the construct shown in panel A (E150Q and L180M). (C) Ethidium bromide-stained agarose gel showing the results of gene-specific PCRs of cDNA synthesized from RNA derived from liver homogenates of a wild-type mouse and a germline transgenic mouse expressing *P. anubis APOL1* from the *ROSA26* locus. (D-F) Kaplan-Meier survival curves of mice infected with 5000 *T. b. brucei* (427-SRA) parasites intraperitoneally (i.p.). (D) Heterozygous or homozygous transgenic mice expressing either *P. hamadryas APOL1* from the *Myosin heavy chain 9* (*Myh9)* locus or *P. anubis APOL1* from the *ROSA26* locus compared to wild-type counterparts. (E) *P. anubis APOL1* and human APOA-I-expressing mice compared to wild-type counterparts. (F) *P. anubis APOL1*-expressing mice expressing *P. hamadryas HPR* by HGD compared to *APOL1* or *HPR* alone (p = 0.09; Log-rank test).

Supplementary Figure 5. Characterization of health with targeted integration of the *P. hamadryas APOL1* cDNA driven by a ubiquitin promoter.

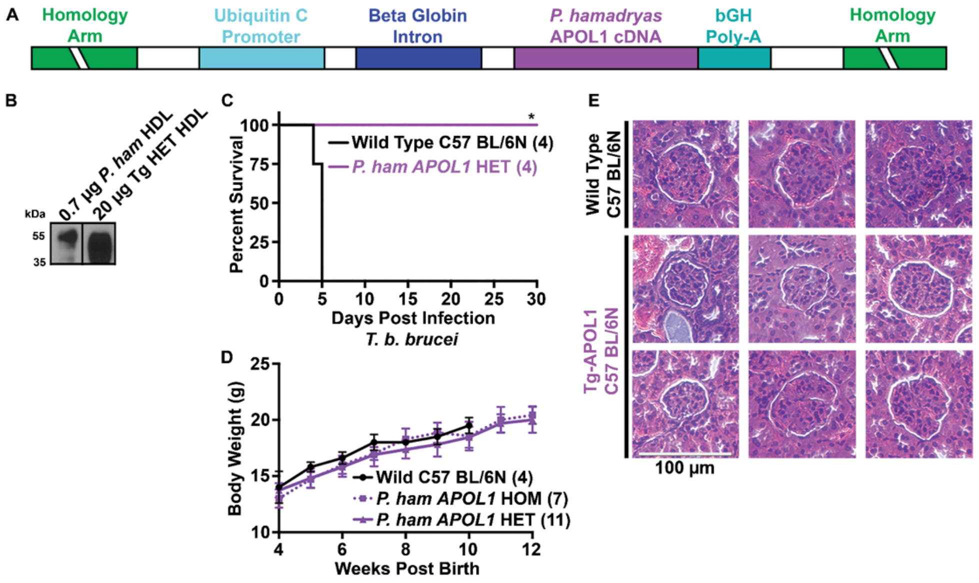

(A) Schematic of the targeting construct used to insert a single copy of the *P. hamadryas APOL1* cDNA into the *ROSA26* locus of the mouse genome. bGH is bovine growth hormone. (B) Anti-baboon APOL1 western blot of purified *P. hamadryas* HDL and transgenic mouse HDL samples. (C) Kaplan-Meier curve showing the survival of *P. hamadryas APOL1* transgenic heterozygous mice compared to wild type counterparts infected with 5000 *T. b. brucei* Lister-427 parasites i.p. (* p = 0.01; Log-rank test). (D) Weight gain in heterozygous and homozygous APOL1 transgenic mice compared to wild type counterparts as a function of time post-birth (error bars represent the mean +/- the SD for each group each week). (E) Hematoxylin and eosin-stained kidney tissue slices focused on a glomerulus from adult wild type and *APOL1* HET transgenic mice. Images represent three mice from each group. Images were captured using a light microscope with 40X magnification.

Supplementary Figure 6. Targeted integration of *P. hamadryas APOL1* and *HPR* is not sufficient to mediate full protection against all trypanosome infections.

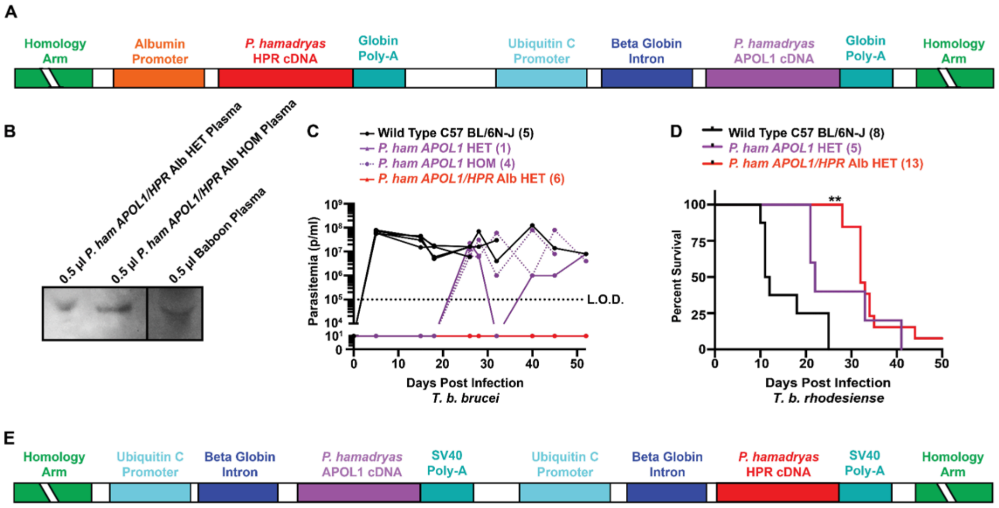

(A) Schematic of the targeting construct used to insert the *P. hamadryas APOL1* and *HPR* cDNAs into the *ROSA26* locus of the mouse genome with *APOL1* expression driven by a ubiquitin promoter and *HPR* by an albumin (alb) promoter. bGH is bovine growth hormone. (B) Western blot showing the relative concentration of HPR in the plasma of heterozygous (HET) and homozygous (HOM) transgenic mice compared to a representative P. hamadryas plasma sample using an anti-human Hp antibody that cross-reacts with human and primate HPR. (C) Parasitemia over time showing the number of parasites per mL of blood in transgenic mice inoculated with 5000 pleomorphic *T. b. brucei* (AnTat 1.1) parasites by i.p. on day 0. The graph shows the parasitemia in each individual mouse of each genotype (number of mice per genotype, including wild type, in parentheses). Parasitemia was counted by light microscopy; the limit of detection is approximately 3-5 x 10^6 parasites per mL (p/mL), denoted by the dashed line. The *P. hamadryas APOL1/HPR* Alb HET cohort are represented schematically as 1, though we did not detect parasites in these mice. (D) Kaplan-Meier survival curve of wild-type and HET transgenic mice inoculated with 5000 *T. b. rhodesiense* (KETRI 243) parasites i.p. (**** $p < 0.0001$; Log-rank test). (E) Schematic of the targeting construct used to insert the *P. hamadryas APOL1* and *HPR* cDNAs into the *ROSA26* locus of the mouse genome with *APOL1* and *HPR* expression both driven by a ubiquitin (ubq) promoter.

Supplementary Figure 7. Chimeric Hum360 APOL1 is associated with HDL in transgenic mice.

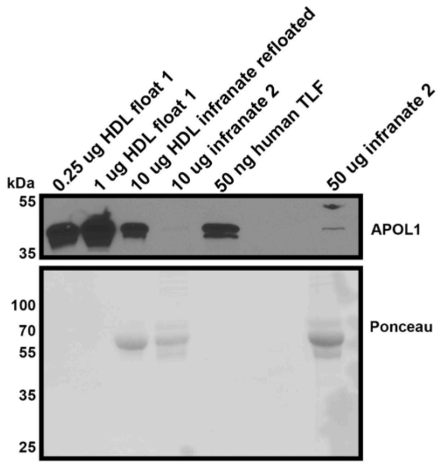

Total HDL lipoproteins were isolated from 4 heterozygous transgenic mice with the Human/*P. hamadrayas* chimeric *APOL1* (Hum360) gene. HDL was isolated by density gradient ultracentrifugation. The top fraction of the first centrifugation step was the total HDL (HDL float 1). The infranate (dense, lipid-poor protein fraction of the lipoprotein isolation) was collected, and floated a second time, with the top fraction being HDL that was missed in the first float (HDL float 2), and the bottom fraction being any non-lipid associated protein (infranate). Each fraction was probed for APOL1 by western blot, with human TLF as a positive control. Ponceau is shown for verification of protein loading in infranate fractions.

Supplementary Figure 8. Validation of protein expression and parasite identity in transiently transgenic mice (hydrodynamic gene delivery).

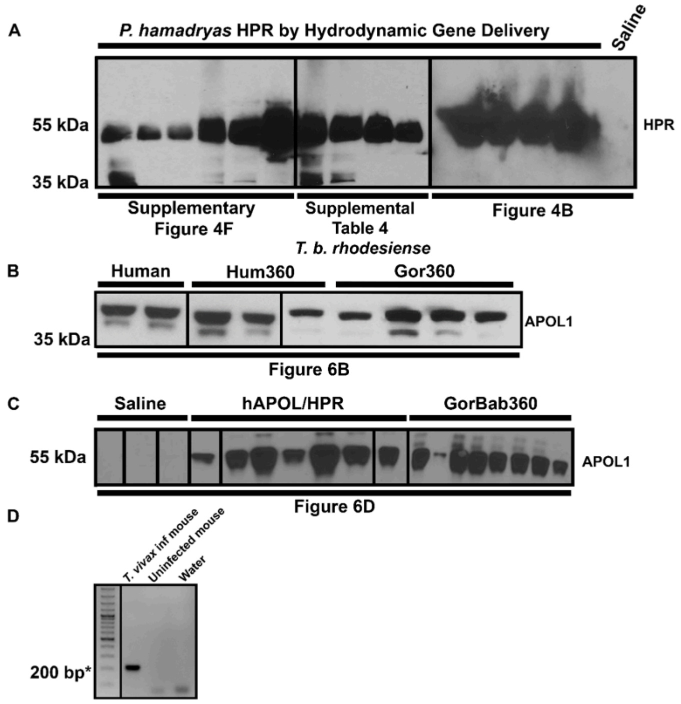

(A-C) The mice expressing either *HPR* (A), or *APOL1* and *HPR* (C), or the *APOL1* chimeras (B, C) via HGD in various experiments were assayed for protein production by western blot of 0.75 µL of plasma collected 2 days-post vector injection by HGD (the same day as parasite infection) to confirm that the data could be interpreted without consideration of poor protein expression. (A) The experimentally transfected mice in the three *HPR*-HGD experiments are shown in the top blot, whereas only one saline / negative control is depicted. (D) *Trypanosoma vivax* (IL1392) identity was confirmed by PCR from DNA extracted from blood of infected and uninfected mice, using established primers. The expected band size was 200 bp, indicated with an asterisk.

Supplementary Figure 9. All raw western blots and Coomassie stains in this manuscript.

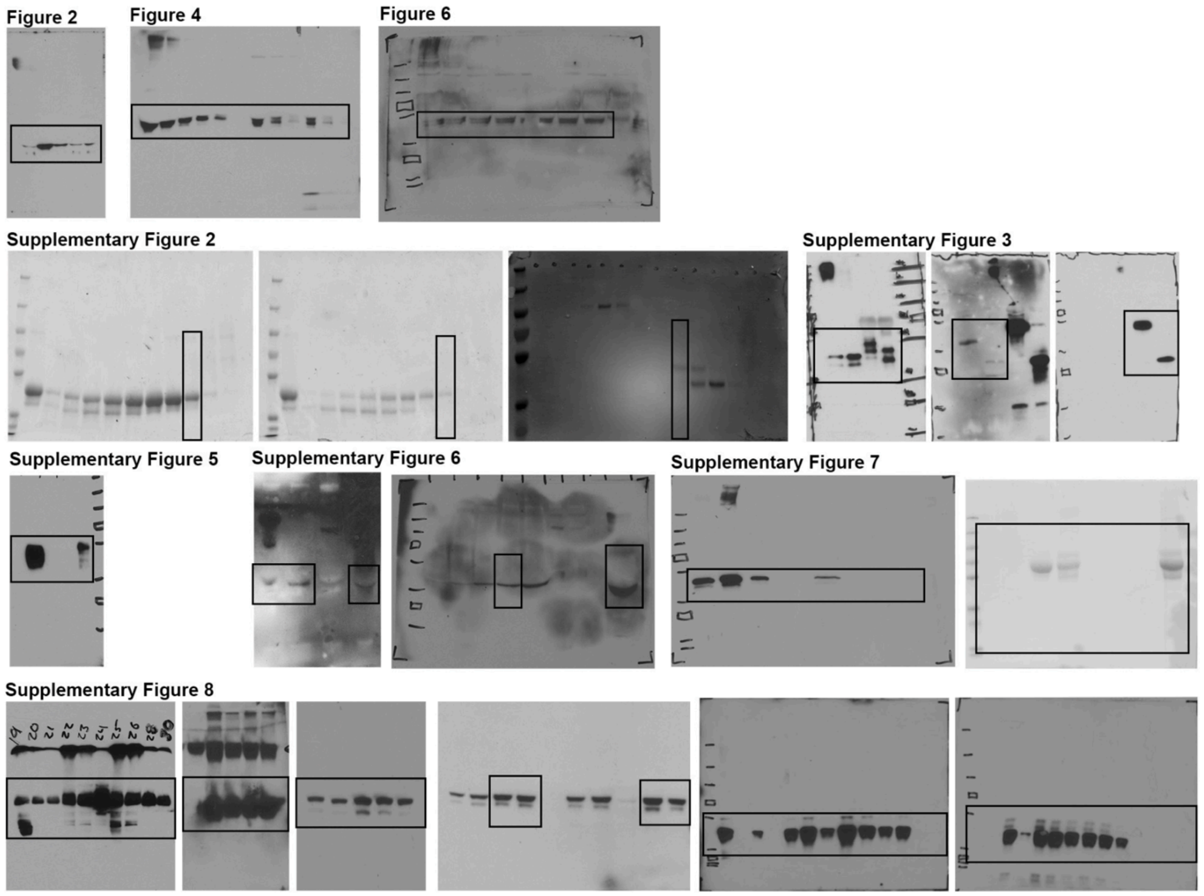

Tables

Supplementary Table 1. Sources of *Papio* DNA used in this study.

Sample ID	Species	Biomaterial Provider Institution/Wild	DNA Source	Sex
L142	Papio anubis	Southwest National Primate Research Center (SNPRC)	WBC	M
LIV5	Papio anubis	Southwest National Primate Research Center (SNPRC)	Liver	F
28697	Papio ursinus	Southwest National Primate Research Center (SNPRC)	N/A	F
28755	Papio ursinus	Southwest National Primate Research Center (SNPRC)	N/A	F
28547	Papio papio	Southwest National Primate Research Center (SNPRC)	N/A	N/A
30388	Papio papio	Southwest National Primate Research Center (SNPRC)	N/A	M
34474	Papio kindae	BCM/Blood collected in the wild - in southwest Zambia	Blood	F
34472	Papio kindae	BCM/Blood collected in the wild - in southwest Zambia	Blood	M
34449	Papio kindae	BCM/Blood collected in the wild - in southwest Zambia	Blood	F
30877	Papio anubis	Yerkes National Primate Research Center (YNPRC) - Aberdare region of Kenya	WBC	N/A
30977	Papio anubis	Yerkes National Primate Research Center (YNPRC) - Aberdare region of Kenya	WBC	N/A
16066	Papio cynocephalus	Baylor College of Medicine - collected in central Tanzania	WBC	F
16098	Papio cynocephalus	Baylor College of Medicine - collected in central Tanzania	WBC	F
97074	Papio hamadryas	Washington University School of Medicine and NYU - Awash Park, Ehtiopia	WBC	F
97124	Papio hamadryas	Washington University School of Medicine and NYU - Awash Park, Ethiopia	WBC	M

Supplementary Table 2. Amino acid sequences of *Papio* APOL1 proteins generated by the observed Indels. Asterisks correspond to those present in Supplementary Figure 1.

Starting from amino acid position 300	
**No Indels**	SVEE---RA----RVVEMERV
**Indel Set 1 (**)**	SVEE-MERVAEMERVVEMERV
**Indel Set 2 (***)**	SVEEEMERVAEMGRVVEMERV

Supplementary Table 3. Analysis of synonymous (dS) and nonsynonymous (dN) substitution rates in *Papio APOL1*. The estimate from the one-ratio model (M0) (dN/dS= 0.5773) is similar to the dN/dS values of immune-related proteins from humans (28). The maximum likelihood analysis of dN/dS ratios by PAML (see Methods) indicates a nearly neutral model (M1a) where the majority (~75%) of nucleotide sites are under purifying selection with the rest of sites under neutral evolution.

Model	t_s/t_v	d_N/d_s	lnL (likelyhood)
M0	6.4427	0.5773	-2001.0488
M1a	5.2927	0 (74.9%); 1(25.1%)	-1988.6290
M2a	5.2927	0 (74.9%); 1 (25.1%); >1 (0)	-1988.6291

Supplementary Table 4. Summary of all transgenic mice parasite survival. The data from various gene constructs and parasite challenges is summarized and expanded here. The parasite challenges presented here are only experiments where survival is the main observation. The genotype is indicated by the color. Data is shown as the number of mice alive over total inoculated. Data from 4 time points is shown to showcase the variability in parasite development for each gene construct. Experiments where the main observation is emergence and sustainability of parasitemia are depicted visually (*T. congolense* in Figure 4 and Figure 5, and *T. b. brucei* Antat 1.1 in Supplementary Figure 6). All data from every challenge is summarized in Table 1.

Mouse Genotype	Parasite (Inoculum)		Alive/Inoculated			
		Day	5	20	35	50
APOL1	*T. b. brucei*	HET	4/4			4/4
	427-SRA (5000)	WT	0/4			0/4
	T. b. rhodesiense	HOM		5/5	5/5	5/5
	KETRI 243 (5000)	HET		5/6	0/6	0/6
		HET + HPR HGD		4/4	4/4	4/4
		WT + HPR HGD		0/6	0/6	0/6
		WT		0/9	0/9	0/9
	T. b. gambiense	HOM	5/5	0/5		
	ELIANE (5000)	WT	0/3	0/3		
	T. b. gambiense	HOM	5/5	3/5	3/5	2/5
	ELIANE (500)	WT	3/3	0/3	0/3	0/3
	T. b. evansi	HOM	4/4			4/4
	AnTat 3 (5000)	WT	4/4			0/4
APOL1/HPR alb	*T. b. rhodesiense*	HET	13/13	13/13	3/13	1/13
	KETRI 243 (5000)	WT	8/8	2/8	0/8	0/8
APOL1/HPR ubq	*T. b. rhodesiense*	HOM	5/5	5/5		5/5
	KETRI 243 (5000)	HET	4/4	4/4		4/4
		WT	4/4	0/4		0/4
	T. b. gambiense	HOM	3/3	0/3		
	ELIANE (5000)	WT	0/2	0/2		
Hum360 APOL1	*T. b. rhodesiense*	HET	4/4	4/4		4/4
	KETRI 243 (5000)	WT	5/5	0/5		0/5
Gor360 APOL1	*T. b. rhodesiense*	HGD	4/4	4/4		4/4
	KETRI 243 (5000)	Human APOL1	6/6	0/6		0/6
	T. vivax	HGD	10/10	3/10	0/10	0/10
	IL1392 (5000)	Human APOL1/HPR	6/6	2/6	1/6	0/6
		Saline	13/13	5/13	3/13	0/13

Supplementary Table 5. Oligonucleotides used for Genotyping of Transgenic Mice by Real-Time PCR.

Oligonucleotides for Genotyping by Real-Time PCR				
**Modification**	**Assay**	**Type**	**Oligonucleotide**	**Sequence**
Baboon APOL1 cDNA into Rosa26 locus	Rosa26-LOA	Loss-of-Allele	Forward Primer	CGTGATCTGCAACTCCAGTCTT
			Reverse Primer	CACACCAGGTTAGCCTTTAAGCC
			Probe	AGATGGGCGGGAGTCTTCTGGGC
	GOA1	Gain-of-Allele	Forward Primer	GGAAAGGGAGCTTCAGGATAAAA
			Reverse Primer	GCGCCTTTGTGGACCTTCT
			Probe	ACCTTGCAGGTCAGG
	Cre-GOA	Floxed Cassette	Forward Primer	TGGTCTGGACACAGTGCCC
			Reverse Primer	TATTGAAACTCCAGCGCGG
			Probe	CCATATCTCGCGCGGCTCCG
	Neo-GOA	Floxed Cassette	Forward Primer	GGTGGAGAGGCTATTCGGC
			Reverse Primer	GAACACGGCGGCATCAG
			Probe	TGGGCACAACAGACAATCGGCTG
Baboon HPR and APOL1 cDNA into Rosa26 locus	Rosa26-LOA	Loss-of-Allele	Forward Primer	CGTGATCTGCAACTCCAGTCTT
			Reverse Primer	CACACCAGGTTAGCCTTTAAGCC
			Probe	AGATGGGCGGGAGTCTTCTGGGC
	GOA2	Gain-of-Allele	Forward Primer	TCCCTGGCAGGCTAAGATG
			Reverse Primer	GGTCAGCAGCCATTGTTCATTG
			Probe	TTCCCGCCATAATCTCACCACAGG
	GOA3	Gain-of-Allele	Forward Primer	TGCAACTCCAGTCTTTCTAGGTAC
			Reverse Primer	CTGAGAGAAGCAGAAGCTTAGGA
			Probe	TTGGATCCAGCATGACGTTCCAC
	Cre-GOA	Floxed Cassette	Forward Primer	TGGTCTGGACACAGTGCCC
			Reverse Primer	TATTGAAACTCCAGCGCGG
			Probe	CCATATCTCGCGCGGCTCCG
	Neo-GOA	Floxed Cassette	Forward Primer	GGTGGAGAGGCTATTCGGC
			Reverse Primer	GAACACGGCGGCATCAG
			Probe	TGGGCACAACAGACAATCGGCTG
Baboon HPR and APOL1, separately driven by Ubiquitin-C promoters, into Rosa26 locus	Rosa26-LOA	Loss-of-Allele	Forward Primer	CGTGATCTGCAACTCCAGTCTT
			Reverse Primer	CACACCAGGTTAGCCTTTAAGCC
			Probe	AGATGGGCGGGAGTCTTCTGGGC
	GOA4	Gain-of-Allele	Forward Primer	AGGGTAGGCTCTCCTGAATCG
			Reverse Primer	CCAAAGAAACTGACGCCTCAC
			Probe	ACAGGCGCCGGACCTCTGGT
	GOA5	Gain-of-Allele	Forward Primer	GCCCACAGGAGATGCTACTC
			Reverse Primer	CCTGGGCAGTTCAGCAGTA
			Probe	TGCTGAGTGACCATAAAGCCTGGG
	GOA6	Gain-of-Allele	Forward Primer	GCTGAGGAGCTGAAGAAGGT
			Reverse Primer	TTGGCGCAGAGTCTCATAC
			Probe	TCAGGAGCTGGAGAAGAAGCTAAACA
	GOA7	Gain-of-Allele	Forward Primer	GGAGCACTTGGTTCGCTAC
			Reverse Primer	CCACTGCTTCTCATTGTTTAAGGT
			Probe	TAAGAGCTACTACAGGCTGCGCA
	GOA8	Gain-of-Allele	Forward Primer	GCAGCCCATACTGAATGAACAC
			Reverse Primer	CATCGCCATAGCAGGTGTCTTC

			Probe	CCTTCTGTGCCGGCATGTCTAAGTA
	Cre-GOA	Floxed Cassette	Forward Primer	TGGTCTGGACACAGTGCCC
			Reverse Primer	TATTGAAACTCCAGCGCGG
			Probe	CCATATCTCGCGCGGCTCCG
	Neo-GOA	Floxed Cassette	Forward Primer	GGTGGAGAGGCTATTCGGC
			Reverse Primer	GAACACGGCGGCATCAG
			Probe	TGGGCACAACAGACAATCGGCTG
Human / Baboon chimeric APOL1 into Rosa26 locus	Rosa26-LOA	Loss-of-Allele	Forward Primer	CGTGATCTGCAACTCCAGTCTT
			Reverse Primer	CACACCAGGTTAGCCTTTAAGCC
			Probe	AGATGGGCGGGAGTCTTCTGGGC
	GOA9	Gain-of-Allele	Forward Primer	GAGCGAGGGTGCAACAAA
			Reverse Primer	ACTGCTCTCTGGGTCCATG
			Probe	CGTTCCAAGTGGGACAGATACTGGA
	GOA6	Gain-of-Allele	Forward Primer	GCTGAGGAGCTGAAGAAGGT
			Reverse Primer	TTGGCGCAGAGTCTCATAC
			Probe	TCAGGAGCTGGAGAAGAAGCTAAACA
	Cre-GOA	Floxed Cassette	Forward Primer	TGGTCTGGACACAGTGCCC
			Reverse Primer	TATTGAAACTCCAGCGCGG
			Probe	CCATATCTCGCGCGGCTCCG
	Neo-GOA	Floxed Cassette	Forward Primer	GGTGGAGAGGCTATTCGGC
			Reverse Primer	GAACACGGCGGCATCAG
			Probe	TGGGCACAACAGACAATCGGCTG
Baboon APOL1 into Rosa26 locus	Rosa26-LOA	Loss-of-Allele	Forward Primer	CGTGATCTGCAACTCCAGTCTT
			Reverse Primer	CACACCAGGTTAGCCTTTAAGCC
			Probe	AGATGGGCGGGAGTCTTCTGGGC
	GOA10	Gain-of-Allele	Forward Primer	GCTACCAAGTCTCCTTCACTCT
			Reverse Primer	TGGCAGCTTCCGTATATGGTTT
			Probe	TGCATTCTGCACACCTGAAGACC
	GOA11	Gain-of-Allele	Forward Primer	AACCGAATAGAGATGGGTGAAGGGA
			Reverse Primer	GCGATTCTTCTTGGCTGGATGT
			Probe	CTGCGATCCACAGCACCACCG
	GOA1	Gain-of-Allele	Forward Primer	GGAAAGGGAGCTTCAGGATAAAA
			Reverse Primer	GCGCCTTTGTGGACCTTCT
			Probe	ACCTTGCAGGTCAGG
	Neo-GOA	Floxed Cassette	Forward Primer	GGTGGAGAGGCTATTCGGC
			Reverse Primer	GAACACGGCGGCATCAG
			Probe	TGGGCACAACAGACAATCGGCTG
Baboon APOL1 into mouse ApoL-Myh9 locus	Myh9-LOA1	Loss-of-Allele	Forward Primer	CAGGCTAGGGTTCCACACAGA
			Reverse Primer	TCTTGTGATGAACATTTTGGGTTATC
			Probe	CTGCTCGCCCAGCC
	Myh9-LOA2	Loss-of-Allele	Forward Primer	AACACAGGGCAGAGATTCCAA
			Reverse Primer	CCGTCTTTTGCCTTTTATTATTTGTA
			Probe	CCCGGTGCTGCGAA
	GOA10	Gain-of-Allele	Forward Primer	GCTACCAAGTCTCCTTCACTCT
			Reverse Primer	TGGCAGCTTCCGTATATGGTTT

			Probe	TGCATTCTGCACACCTGAAGACC
	GOA11	Gain-of-Allele	Forward Primer	AACCGAATAGAGATGGGTGAAGGGA
			Reverse Primer	GCGATTCTTCTTGGCTGGATGT
			Probe	CTGCGATCCACAGCACCACCG
	GOA1	Gain-of-Allele	Forward Primer	GGAAAGGGAGCTTCAGGATAAAA
			Reverse Primer	GCGCCTTTGTGGACCTTCT
			Probe	ACCTTGCAGGTCAGG
	Neo-GOA	Floxed Cassette	Forward Primer	GGTGGAGAGGCTATTCGGC
			Reverse Primer	GAACACGGCGGCATCAG
			Probe	TGGGCACAACAGACAATCGGCTG
Human APOA1 transgenic	GOA12	Gain-of-Allele	Forward Primer	TGTCTCACCCTCCAGCCTAAA
			Reverse Primer	TGTTCGCGCAGCTTGCT
			Probe	AACTGGGACAGCGTGACCTCCACCTT

SI References:

1. Cortez, A. P. *et al.* Cathepsin L-like genes of Trypanosoma vivax from Africa and South America--characterization, relationships and diagnostic implications. *Mol. Cell. Probes* **23**, 44–51 (2009).
2. Rogers, J. *et al.* The comparative genomics and complex population history of Papio baboons. *Sci. Adv.* **5**, eaau6947 (2019).
3. Danecek, P. *et al.* The variant call format and VCFtools. *Bioinformatics* **27**, 2156–2158 (2011).
4. Price, M. N., Dehal, P. S. & Arkin, A. P. FastTree 2--approximately maximum-likelihood trees for large alignments. *PLoS One* **5**, e9490 (2010).
5. Yang, Z. PAML 4: phylogenetic analysis by maximum likelihood. *Mol Biol Evol* **24**, 1586–91 (2007).
6. Lyons, D. M. & Lauring, A. S. Evidence for the Selective Basis of Transition-to-Transversion Substitution Bias in Two RNA Viruses. *Mol. Biol. Evol.* **34**, 3205–3215 (2017).
7. Stoltzfus, A. & Norris, R. W. On the Causes of Evolutionary Transition:Transversion Bias. *Mol. Biol. Evol.* **33**, 595–602 (2016).
8. Thomson, R. & Finkelstein, A. Human trypanolytic factor APOL1 forms pH-gated cation-selective channels in planar lipid bilayers: relevance to trypanosome lysis. *Proc. Natl. Acad. Sci. U. S. A.* **112**, 2894–2899 (2015).
9. Schaub, C. *et al.* Cation channel conductance and pH gating of the innate immunity factor APOL1 are governed by pore-lining residues within the C-terminal domain. *J. Biol. Chem.* **295**, 13138–13149 (2020).
10. Räz, B., Iten, M., Grether-Bühler, Y., Kaminsky, R. & Brun, R. The Alamar Blue assay to determine drug sensitivity of African trypanosomes (T.b. rhodesiense and T.b. gambiense) in vitro. *Acta Trop.* **68**, 139–147 (1997).
11. Genovese, G. *et al.* Association of trypanolytic ApoL1 variants with kidney disease in African Americans. *Science* **329**, 841–845 (2010).
12. Thomson, R. *et al.* Evolution of the primate trypanolytic factor APOL1. *Proc. Natl. Acad. Sci. U. S. A.* **111**, E2130-2139 (2014).

13. Gordon, S. M., Deng, J., Lu, L. J. & Davidson, W. S. Proteomic characterization of human plasma high density lipoprotein fractionated by gel filtration chromatography. *J Proteome Res* **9**, 5239–49 (2010).
14. Valenzuela, D. M. *et al.* High-throughput engineering of the mouse genome coupled with high-resolution expression analysis. *Nat. Biotechnol.* **21**, 652–659 (2003).
15. Pinkert, C. A., Ornitz, D. M., Brinster, R. L. & Palmiter, R. D. An albumin enhancer located 10 kb upstream functions along with its promoter to direct efficient, liver-specific expression in transgenic mice. *Genes Dev.* **1**, 268–276 (1987).
16. Poueymirou, W. T. *et al.* F0 generation mice fully derived from gene-targeted embryonic stem cells allowing immediate phenotypic analyses. *Nat. Biotechnol.* **25**, 91–99 (2007).
